# Relative contributions to vergence eye movements of two binocular cues for motion-in-depth

**DOI:** 10.1038/s41598-019-53902-y

**Published:** 2019-11-22

**Authors:** Martin Giesel, Alexandra Yakovleva, Marina Bloj, Alex R. Wade, Anthony M. Norcia, Julie M. Harris

**Affiliations:** 1 School of Psychology and Neuroscience, University of St Andrews, St Andrews, UK; 2 Department of Psychology, Stanford University, Stanford, USA; 3 School of Optometry and Vision Sciences, University of Bradford, Bradford, UK; 4 Department of Psychology, University of York, York, UK

**Keywords:** Psychophysics, Oculomotor system, Motion detection

## Abstract

When we track an object moving in depth, our eyes rotate in opposite directions. This type of “disjunctive” eye movement is called horizontal vergence. The sensory control signals for vergence arise from multiple visual cues, two of which, changing binocular disparity (CD) and inter-ocular velocity differences (IOVD), are specifically binocular. While it is well known that the CD cue triggers horizontal vergence eye movements, the role of the IOVD cue has only recently been explored. To better understand the relative contribution of CD and IOVD cues in driving horizontal vergence, we recorded vergence eye movements from ten observers in response to four types of stimuli that isolated or combined the two cues to motion-in-depth, using stimulus conditions and CD/IOVD stimuli typical of behavioural motion-in-depth experiments. An analysis of the slopes of the vergence traces and the consistency of the directions of vergence and stimulus movements showed that under our conditions IOVD cues provided very little input to vergence mechanisms. The eye movements that did occur coinciding with the presentation of IOVD stimuli were likely not a response to stimulus motion, but a phoria initiated by the absence of a disparity signal.

## Introduction

**Figure 1:**
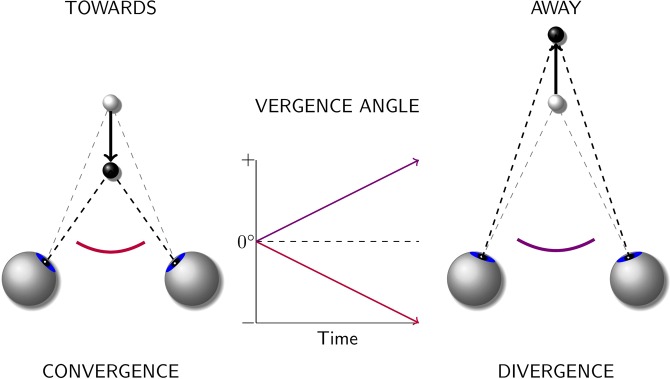
Horizontal disparity vergence and vergence angle conventions used in this paper. Convergence (left) is represented by negative vergence angles and divergence (right) by positive vergence angles. Convergence and divergence refer to horizontal vergence eye movements, whereas towards and away refer to the corresponding stimulus movements triggering the eye movements.

When following an object moving in depth, i.e., an object that moves towards us or away from us, our eyes rotate into opposite directions: if the object approaches, the eyes rotate inwards (convergence), if it recedes, they rotate outwards (divergence). Disjunctive eye movement, in which the eyes move by equal amounts in opposing directions, are referred to as vergence. There are different types of vergence eye movements that are triggered by different cues. These cues include binocular disparity, blur, and the perceived nearness of objects, evoking disparity, accommodative, and proximal vergence, respectively. The two most important cues^1^ identified from previous research are retinal binocular disparity^2^ and blur^3,4^. While vergence eye movements can occur as rotations around three axes – around the interocular axis (vertical vergence), the visual axis (cyclovergence), and an axis perpendicular to the visual and the interocular axes (horizontal vergence)^5^ – when in the following we speak of vergence, we refer only to horizontal binocular disparity vergence. In general, horizontal vergence eye-movements serve to maintain the retinal images on corresponding locations on each eye to enable the fixation of objects on different depth planes (e.g.^5^). Horizontal vergence is quantified by measuring the vergence angle between the eyes (see Fig. 1).

As mentioned above, vergence movements can be triggered by differences between the retinal images in the left and right eye, i.e., by binocular disparities. Disparities are also a potent cue to the perception of depth. By monitoring how disparities change over time (changing disparity, or CD, cue), the visual system can derive information about motion-in-depth. Besides monocular cues, e.g., optic flow and object size change, a second binocular cue — the inter-ocular velocity difference (IOVD) — has been suggested to support the perception of motion-in-depth^6–8^. While the CD mechanism first computes the disparities between the retinal images in the left and right eye and then determines how those disparities change over time, the IOVD mechanism first determines the velocities of the retinal images separately for the left and the right eye and then compares the two resulting monocular velocity vectors. While the two computations are mathematically equivalent^2,8^ and potentially provide similar information about movement in depth, their different orders require separate neural implementations. In real world motion, both types of binocular cues are usually available. Here, we refer to a motion stimulus that carries both CD and IOVD information as the FULL cue stimulus. To selectively probe the effects of CD and IOVD cues, they have to be experimentally separated. This can be done by using random-dot stimuli^9^.

It has been demonstrated that CD information alone allows for the reliable perception of motion-in-depth^6,8,10,11^. Results for IOVD stimuli, however, have been more varied. Whereas some studies found no or only weak use of the IOVD cue^6,12^, others reported that it contributed to speed discrimination, motion after-effects, adaptation, and the discrimination of the direction of motion-in-depth^10,13–26^.

### Previous work on vergence and the IOVD cue to motion-in-depth

Previous studies on vergence eye movements have used a wide variety of different types of stimuli, e.g., isolated light points, lines, gratings or random-dot stereograms. While it is well established that changing disparities trigger vergence eye movements^2,27^ very few studies have investigated vergence eye movements using stimuli that isolate IOVD information^28,29^. To effectively isolate velocity information an IOVD stimulus must provide a consistent monocular motion signal, whilst not generating consistent changes in disparity. There are two methods typically used to construct stereoscopic stimuli that isolate IOVD information (see Methods), either the elements of the stereogram are anti-correlated (aIOVD) between the eyes, i.e., white elements in one eye are matched with black elements in the other, and vice versa, or the elements are spatially de- or un-correlated between the eyes (dIOVD), i.e., an element in one eye does not have a corresponding element in the other eye. The assumptions for using these methods are that in an aIOVD stimulus anti-correlation disrupts the computation of static disparities from paired elements^30–32^ whereas in a dIOVD stimulus disparities cannot be computed because there are no interocular pairs (aside from random pairings). Both methods of isolating IOVD information – as well as the method used to isolate CD information – have shortcomings and neither achieves complete isolation of the targeted cue^7,24,33,34^. In the case of CD isolating stimuli, there might be random temporal correlations^24^. For aIOVD it is not clear whether the disruption of the perception of static disparities also results in a disruption of motion-in-depth perception^35–37^, and in dIOVD stimuli random pairings between dots can potentially introduce a disparity signal^7,38^. By directly comparing the different types of cue-isolating stimuli with each other and with a stimulus that contains both types of information (FULL cue), it is, however, possible to estimate the contributions of the different cues.

Masson *et al*.^28^ measured vergence eye movements with short latencies for human and monkey observers in response to large correlated and anti-correlated random-dot stereograms making small disparity steps in depth. For dense random-dot stereograms (50% dot coverage), they found vergence eye movements in response to both types of stimuli, however, in contrast to the responses to correlated random-dot stereograms, those in response to anti-correlated random-dot stereograms were in the direction opposite to the direction signalled by the change in disparity, i.e., when stimulus motion was consistent with motion towards the observer, the eyes diverged and when the stimuli signalled motion away from the observer, they converged. Reducing the dot density to 7.5% dot coverage, resulted in a decrease of this tendency observed for the anti-correlated stimulus. The eyes first went briefly in the inconsistent direction, but then moved in the opposite, consistent direction.

Sheliga *et al*.^29^ studied vergence eye movements using a novel IOVD isolating stimulus consisting of sinusoidal gratings moving in depth. To remove interocular correlations, i.e., CD cues, the gratings were displaced in every second video frame by 90° in opposite directions in the two eyes so that the interocular phase differences were either 0° or 180°. In response to these stimuli, they observed both horizontal and vertical vergence eye movements with short latencies whose directions were always consistent with the direction of the IOVD signal generated by the stimuli. They also found similar vergence eye movement in response to another type of IOVD isolating stimulus, uncorrelated one-dimensional white-noise stimuli.

Both studies^28,29^ used small numbers of mostly experienced observers. Sheliga *et al*.^29^ tested only the IOVD condition, but to be able to gauge the contribution of the velocity mechanism to vergence eye movement, it is also important to determine how this potential contribution compares to the disparity input to vergence eye movements and how the two cues are combined in the response to a stimulus that contains both velocity and disparity signals. We set out to systematically investigate the effects of CD and IOVD cues on vergence eye movements for a sample of participants that was substantially larger than those used in previous studies^28,29^ investigating this topic and using stimuli similar to those that have been previously employed in the investigation of the perception of motion-in-depth. Most importantly, we wanted to determine whether IOVD provides an input to vergence by measuring vergence for the two types of IOVD isolating random-dot stereograms (aIOVD and dIOVD) typically used in perceptual studies. If IOVD triggers vergence eye movements and both types of stimuli isolate the IOVD cue, then they should elicit vergence of similar strength. The usage of random-dot stimuli that contain both CD and IOVD cues (FULL cue stimulus) will allow us to describe how the two types of cues are combined with respect to vergence eye movements.

To further test the potential contributions of a velocity-based mechanism, we used motion with both ramp and step profiles. The rationale was that since the velocity of a step is undefined (infinite), the performance of a velocity-based mechanism should deteriorate for step motion compared to ramp motion which has a clearly defined constant velocity. For step motion of a random dot pattern, particularly for a large displacement, the motion signals are likely to be much noisier than for a ramp. For example, if a random-dot stereogram moves via a step profile, then, for larger dot density and step size, the direction of the resulting monocular motion signals becomes increasingly ambiguous resulting in matches between dots across frames whose motion direction does not necessarily reflect the global motion direction of the stereogram (for an explanation see the Supplemental Information and Supplemental Fig. S12). The step stimulus, therefore, should favour a CD mechanism, over an IOVD mechanism.

## Results

**Figure 2:**
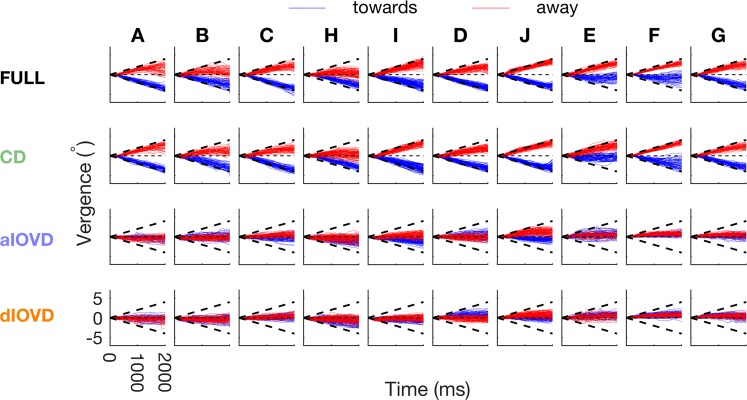
Single trial vergences traces in response to RAMP motion for all conditions separately for each observer. The x-axis shows time in ms (starting from motion onset) and the y-axis shows vergence in degrees. The different stimulus conditions (FULL, CD, aIOVD, dIOVD) are shown in rows and observers in columns. Vergence traces in response to RAMP stimuli consistent with motion towards the observers are depicted in blue, and traces in response to RAMP stimuli consistent with motion away from the observers are in red. Bold black dashed lines indicate stimulus motion towards (negative vergence) and away (positive vergence), respectively.

**Figure 3:**
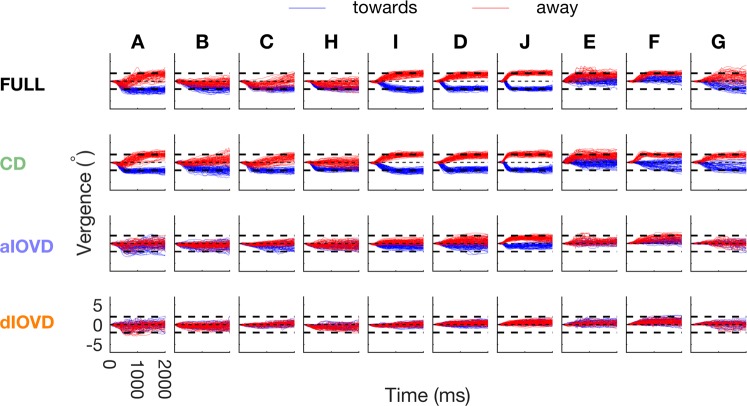
Single trial vergences traces in response to STEP motion for all conditions separately for each observer. The x-axis shows time in ms (starting from motion onset) and the y-axis shows vergence in degrees. The different stimulus conditions (FULL, CD, aIOVD, dIOVD) are shown in rows and observers in columns. Vergence traces in response to STEP stimuli consistent with motion towards the observers are depicted in blue, and traces in response to STEP stimuli consistent with motion away from the observers are in red. Bold black dashed lines indicate stimulus motion towards (negative vergence) and away (positive vergence), respectively.

Raw vergence traces, for all observers, plotting vergence as a function of time are shown for the RAMP stimulus (Fig. 2) and for the STEP stimulus (Fig. 3). Each row shows data for a different stimulus condition. Note first that, even for the FULL cue stimulus (top row), vergence is very different for different observers. For this condition, some observers’ vergence follows the expected pattern for a stimulus moving in depth. For example, for RAMP (Fig. 2), observers I, D and J produced high gain responses that closely follow the stimulus on many trials. Others (e.g., H and E) show high gain for one direction of motion in depth, low for the other, and there are a range of other responses across individuals. In general, vergence traces have higher gain for FULL and CD conditions and are rather poor for aIOVD and dIOVD conditions. For the STEP stimulus for the FULL condition, again, some observers (I, D, J again) show vergence eye movements that closely track the target (Fig. 3), while others do not. Vergence tends to more closely follow the stimulus for FULL and CD, and is poorer for aIOVD and dIOVD, but the pattern is less marked for STEPs than for RAMPs. Note that in the following figures, for ease of comparison, the order in which the observers (A–J) are presented is based on their idiosyncratic vergence biases (see below).

We next conducted detailed analyses to quantify the effects visible in the vergence data. The main analysis is based on the slopes of the vergence traces, as this measure can be used to quantify both RAMP and STEP data. The slope of a vergence trace provides an estimate of the vergence velocity at a given time-point, and the sign of the slope indicates the direction of the eye movement (convergence or divergence). Here, we use the convention that negative slopes indicate convergence, and positive slopes indicate divergence (see Fig. 1). Based on the sign of the slope, we determined the consistency between the direction of the vergence eye movement and the direction of the stimulus movement. By convention, an approaching stimulus has a negative velocity and a receding stimulus a positive velocity. A consistent vergence eye movement is a movement that is in the direction expected for the stimulus movement, i.e., when the stimulus approaches, we expect the eyes to converge, when it recedes the eyes should diverge. In this case, the sign of the slope and the sign of the stimulus velocity are identical (see Methods for details). In addition to the slopes, we also computed the area under the vergence trace. The results of the analysis of vergence movements based on the area can be found in the Supplemental Information.

**Figure 4:**
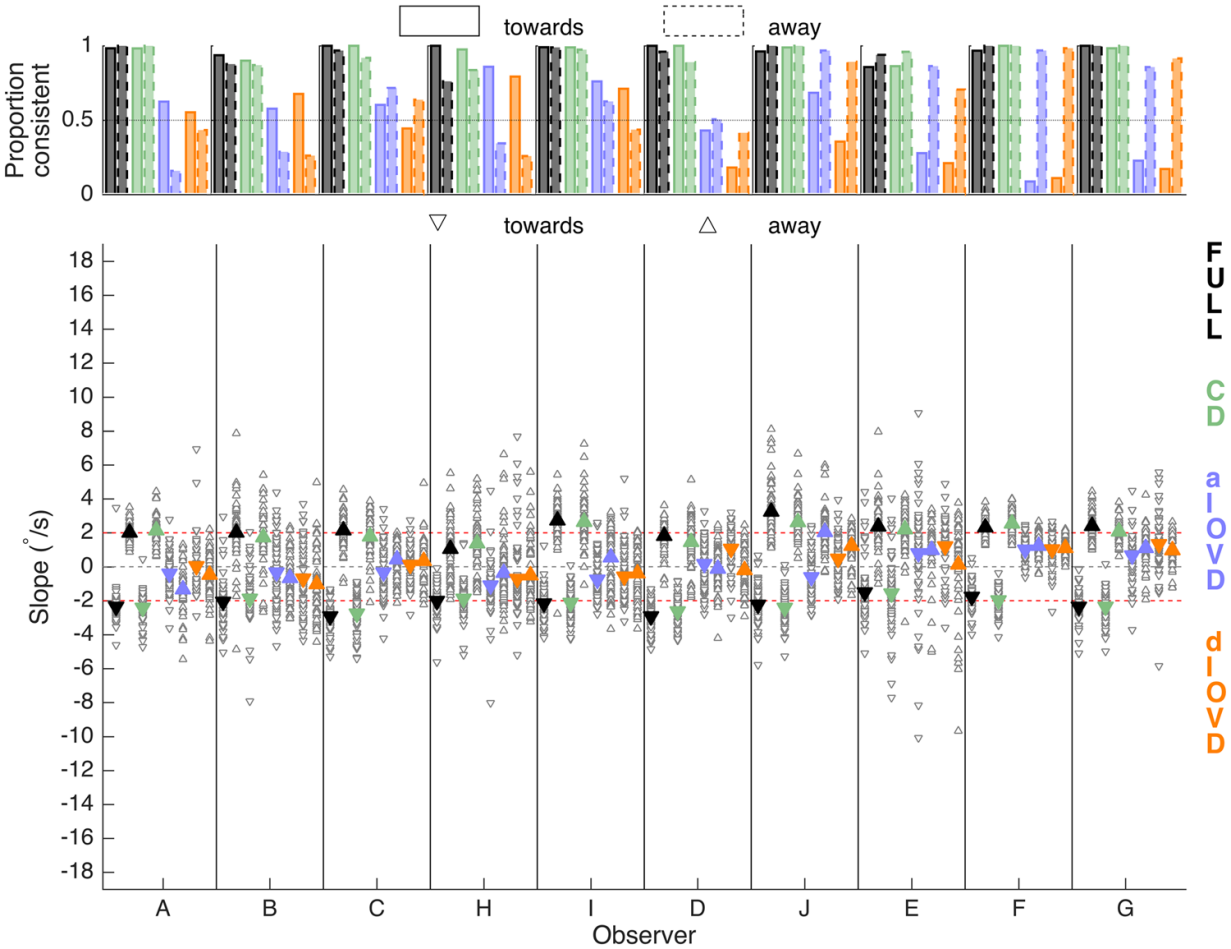
Proportion of consistent vergence slopes (top), slope magnitudes and directions (bottom) for the RAMP condition for each observer, type of motion-in-depth stimulus and motion direction. The x-axis shows the different observers. The symbol/bar colours indicate the stimulus type (black: FULL; green: CD; blue: aIOVD; orange: dIOVD). (Bottom) The y-axis shows the slope of the vergence traces in °/s. Negative slopes indicate convergence, positive slopes divergence. Open grey symbols show slopes of single trials, and filled coloured symbols represent the average over the trials. Upward pointing triangles indicate stimulus motion away and downward triangles stimulus motion towards. The dashed red lines indicate the slope of the RAMP stimulus (±2°/s). (Top) The y-axis shows the proportion of consistent slope directions. Bars with a solid edge represent stimulus motion towards, and bars with a dashed edge represent stimulus motion away.

Figure 4 shows the proportion of consistent vergence responses (upper panel) and slope magnitudes and directions of vergence eye movements (lower panel) for each of 10 participants in response to a stimulus that moved continuously with a constant speed (RAMP). For FULL cue and CD stimuli, the vergence eye movements were almost always in the same direction as the stimulus motion (Fig. 4, top). For some observers this varied depending on the direction of the stimulus movement (observer H). The proportion of consistent slopes was reduced for aIOVD and dIOVD stimuli and exhibited a bias that varied between observers. For example, observers E, F, and G almost always diverged independent of whether the stimulus moved towards or away. Conversely, observer H showed a preference for convergence. The other observers exhibited milder forms of direction biases.

The average slopes for FULL cue stimuli (black triangles, lower panel, triangle orientation indicates convergence – down-facing, or divergence – up-facing) and CD stimuli (green triangles, lower panel) were similar across observers and close to the stimulus velocity (+2°/s for divergence, −2°/s for convergence, red dashed lines in the lower panel). Vergence eye movements for IOVD stimuli (blue for aIOVD, orange for dIOVD) were clearly weaker than those for FULL and CD stimuli, but for most observers they were different from zero and the slopes for aIOVD and dIOVD were similar to each other.

**Figure 5:**
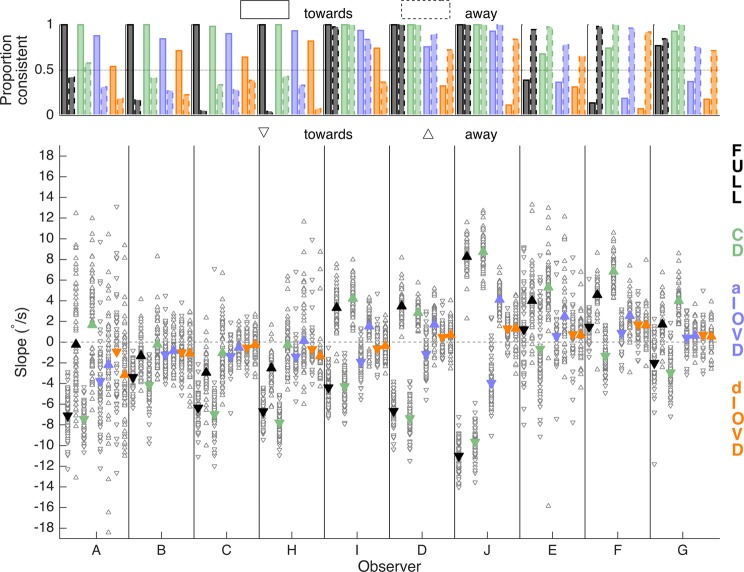
Proportion of consistent vergence slopes (top), slope magnitudes and directions (bottom) for the STEP condition separately for each observer, type of motion-in-depth stimulus and motion direction. The x-axis shows the different observers. The symbol/bar colours indicate the stimulus type (Black: FULL; green: CD; blue: aIOVD; orange: dIOVD). (Bottom) The y-axis shows the slope of the vergence traces in °/s. Negative slopes indicate convergence, positive slopes divergence. Open grey symbols show slopes of single trials, and filled coloured symbols represent the average over the single trial slopes. Upward pointing triangles indicate stimulus motion away and downward triangles stimulus motion towards. (Top) The y-axis shows the proportion of consistent slope directions. Bars with a solid edge represent stimulus motion towards, and bars with a dashed edge represent results for stimulus motion away.

Figure 5 shows single-observer results for STEP motion. There were very different patterns of results for RAMP (Fig. 4) and STEP motion. The most striking was for the proportion of consistent slopes (upper panel) for FULL cue (black bars) and – to a lesser degree – for CD stimuli (green bars). While for RAMPs, the slopes were consistent for the majority of FULL cue and CD stimuli for all observers (Fig. 4), for STEPs most observers (except for I, D, and J) showed far fewer consistent slopes for stimulus movement both away from the observer (A, B, C, H) or towards the observer (E, F, and to a lesser degree G). This demonstrates that STEP stimuli are therefore not consistently driving vergence for FULL cue and CD stimuli. Instead, observers seem to be biased to move their eyes in a direction not driven by the stimulus. We will explore the nature of this bias in the sections below.

The proportion of consistent slopes for IOVD stimuli (blue and orange bars) showed a bias similar to the one for RAMPs. For all observers, the bias that emerged for FULL cue and CD stimuli with STEP motion was consistent with the bias found for IOVD stimuli for RAMP and STEP motion. For three observers (I, D, J), FULL and CD performance for STEPs did not differ from the performance for RAMPs. For those observers, the proportion of consistent slopes for aIOVD stimuli (blue bars) increased for STEPs compared to RAMPs.

**Figure 6:**
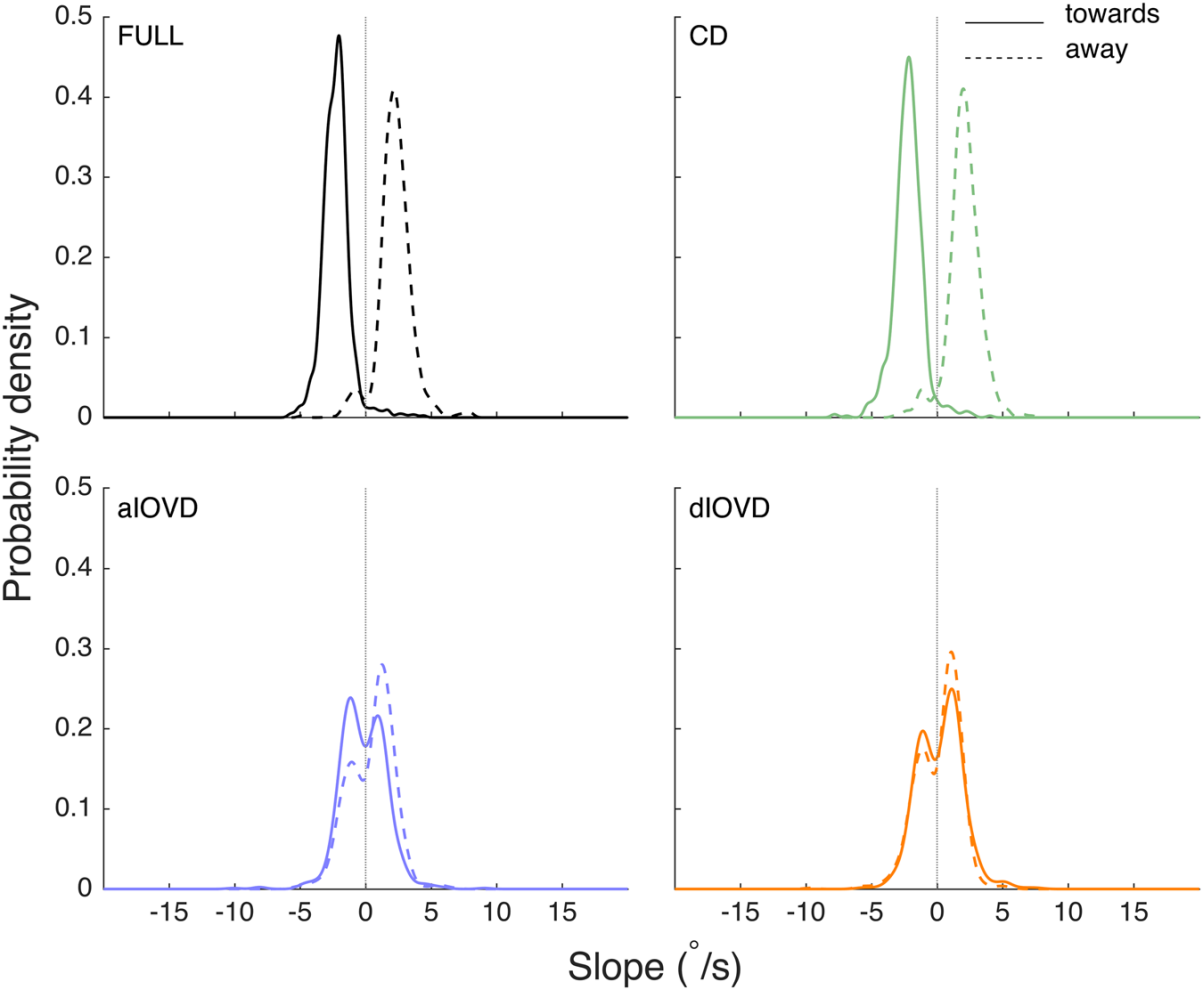
Slope probability density estimates for RAMP motion stimuli. The x-axis shows the slope in °/s, and the y-axis the probability density. The slopes from all observers and trials have been pooled. Negative slopes represent convergence, positive slopes divergence. Distributions with a solid outline represent stimulus motion towards, and distributions with a dashed outline represent stimulus motion away. The total number of slopes differed between conditions.

**Figure 7:**
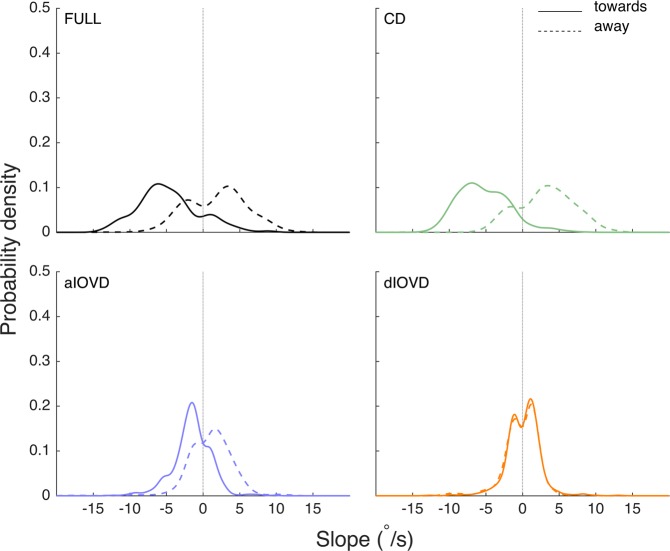
Slope probability density estimates for STEP motion stimuli. The x-axis shows the slope in °/s, and the y-axis the probability density. The slopes from all observers and trials have been pooled. Negative slopes represent convergence, positive slopes divergence. Distributions with a solid outline represent stimulus motion towards, and distributions with a dashed outline represent stimulus motion away. The total number of slopes differed between conditions.

In general, slope magnitudes for FULL cue (black) and CD (green) stimuli were higher for STEPs than for the RAMP stimulus, particularly for stimuli moving towards the observers. Slope magnitudes for IOVD stimuli (blue, orange) were similar to those for the RAMP stimulus. Given that there was a lot of variability in the slope data both within and between observers, using averaged data to make general statements seems of limited validity. So, as a next step, we therefore explored the probability density estimates of the slopes pooled over all individual traces for all observers (the open grey triangles in Figs. 4 and 5, see also Supplemental Table S1). Figures 6 and 7 show the slope probability density estimates, the frequency of each slope estimated from the pooled data, for RAMPs and STEPs, respectively. Probability density estimates were computed using a Gaussian kernel.

For RAMP motion, the towards (solid lines) and away (dashed lines) distributions for FULL cue and CD were clearly separated and unimodal, suggesting a clear separation of vergence responses. The towards distribution was shifted to negative slope values and the away distribution to positive slope values. The overlap between distributions represents slopes that were inconsistent with the stimulus. The overlap for FULL cue and CD was small indicating that most vergence eye movements in these conditions were consistent with the stimulus motion. However, for both types of IOVD stimuli, the distributions were centred on zero and tended to be bimodal, and the towards and away distributions almost completely overlapped. This indicates that the direction of the vergence eye movements in response to IOVD stimuli was largely independent of the direction of the stimulus movement.

For STEP motion (Fig. 7), the distributions for FULL cue and CD were more spread out and had a larger overlap than for RAMP motion. This indicates an increased number of inconsistent vergence movements for the STEP condition. While the slope distribution for dIOVD is similar to the corresponding distribution for RAMP motion, for aIOVD stimuli the overlap between towards and away distributions was slightly reduced indicating a small increase in consistency.

**Figure 8:**
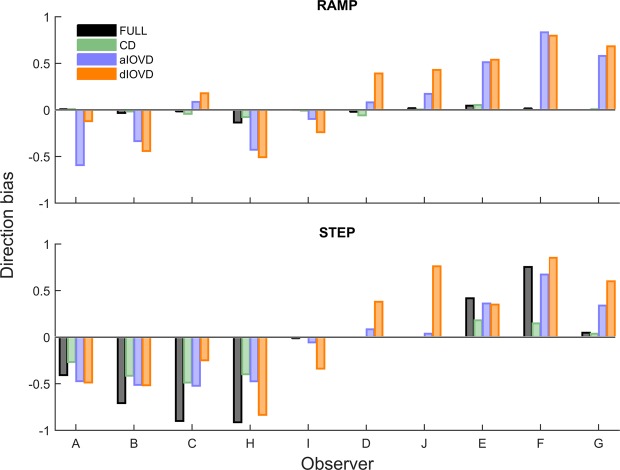
Direction bias for RAMP (top) and STEP (bottom) motion. The x-axis shows the observers, and the y-axis the direction bias. A negative bias indicates a preference for convergence, and a positive bias indicates a preference for divergence. Different bar colours represent the different types of motion-in-depth stimuli (Black: FULL; green: CD; blue: aIOVD; orange: dIOVD). See text for how the bias was computed.

The overlapping slope distributions for IOVD stimuli could indicate that observers’ vergence eye movements in response to IOVD stimuli were random, however, the individual observers’ data reveal that most observers were biased either towards convergence or divergence. To analyse the individual observers’ convergence or divergence biases, we computed for each observer a direction bias (Fig. 8) as (P_away_ − P_towards_)/(P_away_ + P_towards_) where *P* represents the proportions of consistent slope directions for towards and away stimulus movements separately for RAMP and STEP motion.

For RAMP motion (top), vergence direction biases largely occurred only for IOVD stimuli (blue, orange). Roughly, there were two groups of observers, one with a convergence (A, B, H, I) and the other with a divergence bias (D, J, E, F, G). For STEP motion (bottom), direction biases also occurred for FULL cue and CD stimuli for six out of ten observers. The direction of the bias for IOVD stimuli was consistent for RAMP and STEP motion for all observers except for observer C. If a bias for FULL and CD stimuli occurred for STEP motion, its direction was the same as the direction of the bias for IOVD stimuli. Four observers (I, D, J, G) remained unbiased for FULL cue and CD stimuli. Of those, observers I, D, and J also hardly showed any bias for aIOVD stimuli both for RAMPs and STEPs.

**Figure 9:**
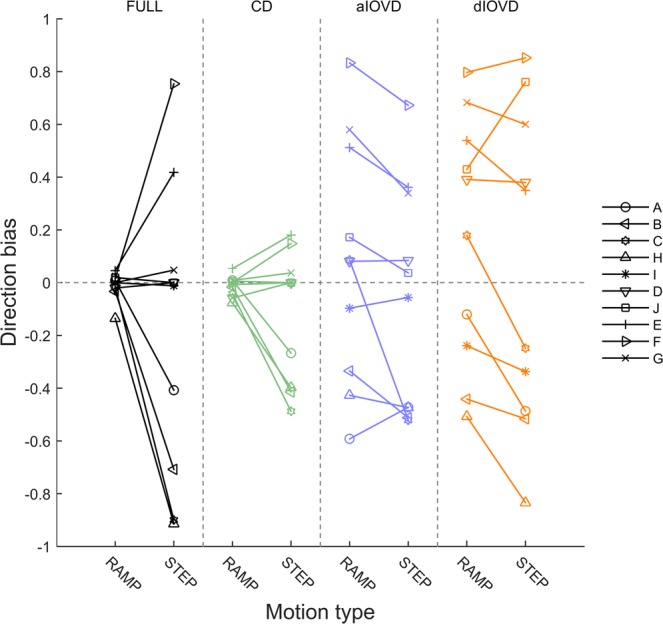
Direction bias for RAMP and STEP motion. The x-axis shows the motion type (RAMP and STEP) separately for each stimulus type in separate columns (Black: FULL; green: CD; blue: aIOVD; orange: dIOVD). The y-axis shows the direction bias. A negative bias indicates a preference for convergence, and a positive bias indicates a preference for divergence. Individual lines represent different observers identified by the symbols at the endpoints of the lines. See text for how the bias was computed.

In Fig. 9 the direction bias is plotted in a way that emphasises the changes in the bias depending on the type of stimulus motion. Each panel shows data for one motion type. On each, lines connect data points for the RAMP and STEP conditions for each observer. While for FULL cue and CD stimuli (two left columns) biases for RAMP motion were small, the biases increased sharply for FULL cue and moderately for CD stimuli for STEP motion. In contrast, for IOVD stimuli (right two columns) the biases were already large for most observers for RAMP motion and there was no consistent increase or decrease for STEP motion.

## Discussion

We investigated vergence eye movements using stimuli that isolated or combined two different binocular cues to motion-in-depth. Our goal was to determine the extent to which IOVD cues drive the vergence system, under stimulus conditions that are typical of vision experiments investigating the perception of motion-in-depth using these kinds of stimuli. We used stimuli that had a ramp motion profile and stimuli with a step motion profile with the aim of potentially isolating a velocity-based mechanism. For RAMP motion, we found that stimuli containing disparity information (FULL, CD) reliably elicited vergence eye movements. These eye movements were largely similar both in terms of the magnitude and consistency for almost all observers. For stimuli that were designed to contain only velocity information (aIOVD, dIOVD), vergence eye movements were weaker and, more importantly, independent of the direction of the stimulus motion. Most observers exhibited a convergence or divergence bias for either one of the two types of IOVD stimuli or for both. These results for RAMP motion strongly suggest that only the CD cue, not the IOVD cue, triggers vergence eye movements that are consistent with the stimulus motion under the conditions we used.

The findings for step motion corroborated these conclusions regarding the IOVD stimuli. If the IOVD cue contributed to vergence eye movements, performance for aIOVD and dIOVD should have deteriorated in the STEP condition because the step motion stimulus does not contain a reliable velocity signal. We found that the changes in performance for IOVD stimuli were not consistent with this prediction. For IOVD stimuli, performance was similar for STEPs and RAMPs. There was no consistent decrease in magnitude or consistency for IOVD stimuli for STEP motion. This is particularly true for the dIOVD stimulus. For the aIOVD stimulus, an increase in the consistency of the vergence eye movements was found especially for observers I, D, and J. This indicates that dIOVD and aIOVD might differ in the effectiveness with which they isolate the IOVD mechanism (see below). Overall, the findings for the IOVD stimuli suggest that the IOVD cue itself did not trigger vergence eye movements in our experiments. We speculate that the eye movements observed in response to the presentation of IOVD stimuli were the result of a phoria being initiated by the absence of a fusable stimulus (by their design IOVD stimuli do not have elements that can be fused between the two eye views)^39^. Some of the observers exhibited an esophoria (convergence bias) and others an exophoria (divergence bias). Except for one observer (C), the direction of the bias was consistent across cue and motion types.

Our analysis of vergence eye movements focused on the slope and direction (con-/divergence) of the eye movements because the computations for these two parameters are straightforward and relatively robust when applied automatically to single vergence traces. Nevertheless, for vergence traces that show non-monotonic behaviour in their early stages, i.e., mostly those in response to IOVD stimuli, the automatic computation of the slope might occasionally fail. As a control, we, therefore, also computed the area under the vergence trace which is less susceptible to these early changes in the direction of the eye movement since it was based on the integration over the complete vergence trace (2 s duration) and is dominated by the later components of the vergence movement. Supplemental Figs. S5–S10 (corresponding to Figs. 4–9) show the results based on the area under the vergence trace. Both measures lead to very similar conclusions. One advantage using the area under the trace is that in our case the RAMP and the STEP stimuli had the same area allowing a direct comparison of the vergence traces for RAMPs and STEPs. Supplemental Figs. S5 and S6 show that vergence traces for RAMPs and STEPs have similar areas under the vergence traces.

Qualitatively analysing the shapes of the vergence traces provides further corroborating evidence against a role of IOVD in triggering vergence eye movements. Supplemental Figs. S4 and S11 show the averaged trajectories for each observer. In Fig. S4 the criterion for the identification of in-/consistent trajectories was based on the sign of the slope whereas the classification in Fig. S11 was based on the sign of the area under the trajectory. Vergence traces for RAMPs and STEPs have distinctly different shapes^40^ which can be clearly seen for FULL cue and CD stimuli for consistent vergence traces (Supplemental Figs. S4/S11, first and second rows, first and last columns). For inconsistent vergence traces the shapes were less distinct (first and second rows, middle two columns). For the IOVD stimuli (third and fourth rows), with only a few exceptions for aIOVD, there were hardly any differences in the shapes of consistent and inconsistent vergence traces for RAMP or STEP stimuli. Thus, the vergence movements in response to the presentation of IOVD stimuli seem less likely to be stimulus specific. The similarity of the traces is consistent with the suggestion that they are merely a reaction to the absence of a fusable stimulus.

For FULL cue and CD stimuli, the STEP stimulus consistently failed to drive vergence, particularly for some observers. Inconsistencies between the direction of random-dot stereograms moving in depth and vergence eye movements have been reported previously. Busettini *et al*.^39^ found that for large correlated random-dot stereograms vergence movements were always in the expected direction for small disparity steps (<2°—3°) but for larger disparity steps (12.8°) eye movements became independent of the direction of the disparity steps with participants exhibiting preferred directions. They compared these preferred directions with the directions of the participants’ steady-state phorias and found that the preferred directions in response to large disparity steps were often not consistent with the participants’ phorias. They attributed these inconsistent vergence movements to the observers making “globally ‘false’ matches” between the random-dot stereograms. While it is indeed possible that such false matches between the dots in the two eyes could explain the increase in inconsistent vergence movements for FULL cue and CD stimuli in the STEP condition, the consistency found in our data in the direction of the ‘false’ vergence movements for each observer across different types of stimuli and movement types seems to make this a less likely explanation. For the IOVD stimuli, inconsistent vergence eye movements already occurred for RAMP motion – for which globally false matches are less likely to occur – and the preferred directions of the inconsistent movements did not change between RAMPs and STEPs. Where inconsistent vergence movements occurred for FULL cue and CD stimuli, their preferred directions were for most observers consistent with those for the IOVD stimuli. Additionally, the above-mentioned differences in the shapes of consistent and inconsistent traces (Supplemental Fig. S4) also speak against a strong influence of globally false matches, since in that case the vergence movements would have been in directions opposite to the global motion but still should have exhibited the characteristics of vergence traces in response to step and ramp motion, respectively, but this was largely not the case.

One explanation for the surprising finding that some observers (I, D, and J) were largely unbiased for aIOVD stimuli in contrast to dIOVD stimuli and that their performance for aIOVD improved for STEPs compared to RAMPs, could be that these observers might have been able to use the disparity signal that is present in aIOVD stimuli. This has been suggested in the perceptual domain as an explanation for the similar motion-in-depth discrimination performances found for aIOVD and FULL cue stimuli^17,41^. While the perception of motion-in-depth is based on relative disparity, vergence is driven by absolute disparity^42,43^. Absolute disparity is encoded by neurons in V1^44^ and it has been shown that in V1 neurons sensitive to disparity respond to anti-correlated signals^35–37^ with an inverted tuning curve. Consistent with this, Masson *et al*.^28^ reported that for jumps in disparity, large anti-correlated random-dot stereograms elicited vergence eye movements in the direction opposite to the stimulus movement. We did not find a systematic inversion of the direction of vergence eye movements for aIOVD stimuli, but the differences between aIOVD and dIOVD stimuli, found for some observers, suggest that the aIOVD stimuli might be less effective in isolating the velocity signal than the dIOVD stimulus and might contain a consistent disparity signal which can be accessed by the vergence system.

To test whether the different types of random-dot stereograms generated consistent motion direction signals, observers F and J participated in a perceptual control experiment in which they were presented with the same types of random-dot stereograms (FULL cue, CD, aIOVD, dIOVD) and motion types (RAMP and STEP) as used in the main experiment in the same setup but moving laterally (leftwards or rightwards) instead of moving in depth. The observers had to indicate in which direction the stereograms moved. No eye movements were recorded. The results are shown in Supplemental Fig. S12. Unsurprisingly, both observers performed at chance for CD stimuli which by design should not contain a consistent monocular motion signal. For RAMPs, observer J almost always and observer F mostly identified the direction of lateral motion correctly for FULL cue, aIOVD and dIOVD random-dot stereograms. For STEPs, however, both observers performed near chance level for all stimuli. This supports the assumption that step motion does not provide a reliable monocular motion signal. So, both observer J’s accurate perceptual performance (see below) and their largely consistent vergence eye movements for FULL cue and aIOVD stimuli moving in depth – even in the STEP conditions – probably relied on a disparity signal to which observer J might be especially sensitive.

Results from perceptual studies indicate that the sensitivity of the CD mechanism is highest for slow speeds whereas the IOVD mechanism is more sensitive to higher speeds^17,24,45^. The change from RAMP to STEP motion, therefore, might have had a deteriorating effect on the disparity cue as well, and this could explain the decrease in consistency found for CD stimuli for most observers in the STEP condition. The similarity of the magnitudes and consistencies of CD and FULL cue stimuli in the RAMP condition suggests that vergence for the FULL cue stimulus was dominated by the disparity cue. With respect to the consistency, the FULL cue stimulus was more effected by the change to step motion than the CD stimulus and became more similar to the IOVD stimuli. This might point to an adverse effect of the velocity signal in the FULL cue stimulus once the disparity stimulus becomes less reliable. The increase in consistency for aIOVD stimuli from the RAMP to the STEP condition observed for observers D, I, and J, however, cannot be straightforwardly explained.

Sheliga *et al*.^29^ observed short-latency vergence (≈80–90 ms) in response to IOVD stimuli. We did not systematically analyse the latencies of our vergence traces but based on visual inspection they were considerably longer (≈250–300 ms for FULL cue and CD stimuli) and closer to latencies found in a range of other previous studies investigating vergence eye movements with different types of stimuli (e.g.^2,46–49^). For IOVD stimuli, vergence movements often started as soon as the fixation marker was removed.

Most importantly, Sheliga *et al*.^29^ reported that the vergence eye movements in response to their IOVD stimuli were always in the direction signalled by the IOVD cue. This is inconsistent with our results. The most obvious difference between their study and our study are the different types of stimuli that were used. Sheliga *et al*. tested vergence eye movement in response to their novel grating stimulus and to a one-dimensional noise stimulus which they do not describe in detail but mention that it was similar to the type of stimuli used by, e.g., Shioiri *et al*.^25^. Broadly, these stimuli consist of horizontal bands of noise and uniform bands alternating in counterphase in the two eyes so that a band of noise in one eye is always paired with a uniform band in the other eye and vice versa. It has been pointed out that stimuli of this kind could potentially create disparity signals along the boundaries of the bands^50,51^. It could be speculated that similar signals could arise from the novel grating stimulus used by Sheliga *et al*., but their stimuli were not described in enough detail in their paper to be sure of this.

Sheliga *et al*. noted that in IOVD isolating random-dot stereograms random local matches between dots in the two eyes can potentially occur that then might generate a disparity signal in the same direction as the IOVD signal. While it is indeed possible that there could have been spurious local matches between dots in our dIOVD stimulus, they certainly did not result in vergence consistent with the stimulus movement, but even if these matches created a random disparity signal, it would be difficult to explain how this signal could account for inter-individually different but intra-individually consistent vergence biases across stimulus and motion types.

We also asked participants to indicate in each trial by keypress whether they perceived the random-dot stereogram as moving toward or away from them. The analysis of the perceptual responses shows that overall observers — with the notable exception of observer J — were not good at discriminating the direction of motion-in-depth for any of the stimuli (Supplemental Figs. S13 and S14). Since our stimulus did not contain a stationary reference (apart from the frame of the monitor) that could have been used to compute relative disparities, on which the perception of motion-in-depth relies, the weak discrimination performance might not be surprising^42^. The correlation between vergence consistency and proportion of correct discrimination of the direction of motion-in-depth, was low (Supplemental Figs. S15 and S16). These findings are consistent with previous studies that have found no consistent relationship between vergence eye movements and the discrimination of the direction of motion-in-depth^52,53^.

## Conclusions

We found that random-dot stereograms that provide only velocity information (IOVD) do not trigger consistent vergence eye movements. The presentation of IOVD isolating stimuli resulted in eye movements that were weak and largely not indicative of the direction of the stimulus movement and likely represented a phoria resulting from the absence of a fusable stimulus.

## Methods

### Stimuli and set-up

Random-dot stereograms were used in order to eliminate changing size cues and other monocular depth cues such as differential blur that are associated with movement in depth. Random-dot stereograms also facilitated the isolation of the CD and IOVD cues^9^. The random-stereograms were presented on a LG OLED TV (55EF950V-ZA) monitor (123 × 72 cm, 1920 × 1080 pixel, 60 Hz). Stereoscopic presentation was achieved by using the line-interleaved top-down stereo-mode. Observers wore polarized glasses and the experiment was carried out in a darkened room.

The stereograms were comprised of black (≈0.5 cd/m^2^) and white dots (≈158 cd/m^2^) with anti-aliased edges, presented on a mid-grey background (≈75 cd/m^2^) covering a 36° diameter portion of the display. We created four different types of random-dot stereograms that combine or isolate the two binocular cues: FULL cue, CD, aIOVD, and dIOVD. FULL cue refers to the condition in which CD and IOVD information were both present. In the CD random-dot stereograms, correlated dots in the left and right eye were randomly repositioned every 3 video frames, thus update was at 20 Hz. The aIOVD (anti-correlated IOVD) and dIOVD (de-correlated or uncorrelated IOVD) conditions were used to isolate IOVD information as they are supposed to preclude calculation of the CD cue. See Supplemental Fig. S1 and additional descriptions in the Supplemental Information for detailed information about the generation of the random-dot stereograms. The random-dot stereograms were viewed from a distance of 95 cm. The individual dots had a diameter of 0.25°. There were ≈1323 dots in each monocular field, resulting in a dot coverage of 6% in each monocular field.

**Figure 10:**
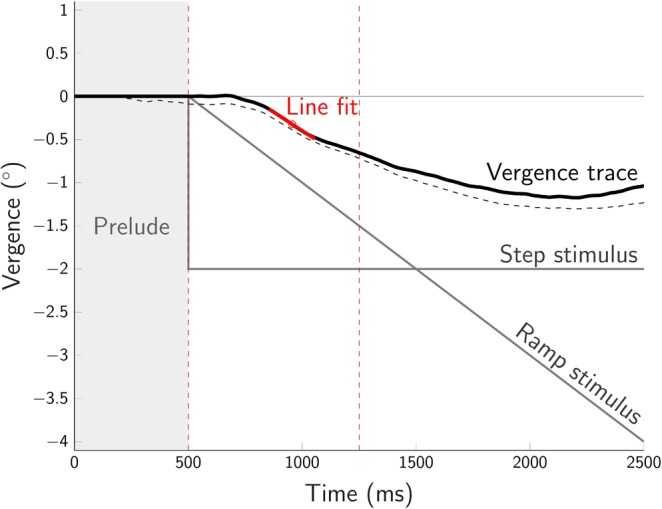
Schematic representation of STEP and RAMP stimulus trajectories (towards), vergence trace (convergence) and data analysis. The x-axis shows the time in milliseconds, the y-axis the vergence angle in degrees. Each trial started with a prelude of 500 ms during which the stimulus did not move in depth. The RAMP stimulus moved continuously for 2 s with a monocular speed of 1°/s. The STEP stimulus made a monocular step of 1°. For data analysis, we calculated the vergence slope thus: the midpoint between the minimum and maximum vergence values within the first 750 ms of the vergence trace (vertical dashed red lines) was determined, and a straight line with a length of 100 ms was fit through this point to the vergence trace. The dashed black curve represents a measured vergence trace, and the continuous black curve shows the same vergence trace after subtraction of the vergence offset at the end of the prelude and setting the prelude vergence to zero.

Each type of random-dot stereogram was tested in two image displacement conditions commonly use to elicit vergence eye movements: RAMP and STEP (see Fig. 10). In the RAMP condition, the random-dot stereograms moved continuously with a monocular speed of 1°/s for 2 s either towards or away from the observer. In the STEP condition, the stimulus made an instantaneous step of 1° (monocular) either towards or away from the observer and remained at that position until the end of the trial after 2 s. For FULL cue, CD, and aIOVD stimuli, the movement started at 0° disparity. For dIOVD stimuli, disparity is undefined.

Each trial started with a period of 500 ms (prelude) in which a version of the random-dot stereogram that did not move in depth was shown, i.e., for FULL, aIOVD, and dIOVD the random-dot stereograms were static, while for CD stimuli correlated dot pairs were randomly repositioned at the rate of 20 Hz without changing disparity. During the prelude FULL cue, CD, and aIOVD stimuli remained at zero disparity. During this period, vertical nonius lines were presented in the centre of the random-dot stereograms. They had a height of 3° and a width of 0.25° and were positioned 0.5° above and below the centre of the screen. Once the stimulus started to move, the nonius lines disappeared.

Eye movements were tracked using a head-mounted SR Research Eyelink^®^ II eye tracker (250 Hz, binocular tracking of corneal reflection). The experiment was programmed and run using MATLAB^54^ together with the Psychophysics Toolbox^55–57^ and the Eyelink Toolbox^58^.

### Procedure

In the first session, the observers’ stereo vision was tested with the TNO test (pass-fail criterion 120″ of retinal disparity). The observers were instructed that they would see black and white moving dots and that their task would be to decide whether the dots moved towards or away from them by pressing one of two keys on a keyboard. After this instruction, the observers were given the opportunity to familiarize themselves with the experiment by running a few test trials without the eye tracker. Then the eye tracker was placed on the observer’s head, the cameras were adjusted, and the calibration and validation procedures were run. If necessary, the procedure was repeated until a valid calibration could be obtained. To reduce head movements, the observers’ head position was stabilized using a chin rest.

Each trial started with a uniformly grey screen with a white fixation cross in the centre of the screen which the observers were instructed to fixate. The observers initiated the stimulus presentation by pressing a key on a keyboard once they were ready. Subsequently the prelude version of the stimulus was shown for 500 ms. The observers were instructed to maintain fixation in the centre between the nonius lines and to try to align the lines. After 500 ms, the nonius lines disappeared and concurrently the stimulus started to move either towards or away from the observer. After the end of the motion, a uniformly grey screen with a message asking the observers to respond either’towards’ or’away’ was displayed. The observers were asked to try not to blink during the stimulus presentation.

The four different types of motion-in-depth stimuli (FULL, CD, aIOVD, dIOVD) and the two motion directions (towards, away) were pseudo-randomly interleaved in each experiment. The different motion conditions (RAMP, STEP) were tested in separate experiments. In general, observers did one STEP and one RAMP experiment per session. In some cases, difficulties in tracking the observers’ eyes allowed only one experiment per session to be completed. The order of STEP and RAMP experiments was pseudo-randomized for each observer. Each experiment consisted of 20 trials for each of the four types of motion-in-depth stimuli and two motion directions resulting in 160 trials per experiment. A single experiment took between 15 and 20 minutes, and the duration of one experimental session was approximately one hour. Before each experiment the calibration and validation procedure of the eye tracker was run. At no point during the experiment did the observers receive any kind of feedback.

Observers participated in at least four sessions. Depending on the number of rejected trials, STEP and/or RAMP experiments were added as needed to reach a minimum of 40 usable trials per condition. Observers participated maximally for eight sessions. Observers who after eight sessions did not have at least 40 usable trials for each condition were excluded from the data analysis.

### Observers

We aimed to test 20 observers. Of those, 10 observers did not complete the experiment for various reasons, e.g., no-shows, eyes not consistently trackable. For four of these 10 excluded observers, no or only incomplete data sets could be recorded. For six other observers at least 40 trials per condition could be recorded, but, in the end, they had too many rejected trials (for some or all conditions) so that they were excluded from the data analysis. Ten observers (seven females) were included in the data analysis. They had varying numbers of completed and usable trials (≥40 trials for each condition). Supplemental Figs. S2 and S3 show the number of completed and usable trials for each observer and condition. Except for observer F, who is one of the authors, all observers were volunteers and naive to the purpose of the experiment. Observer J had previously participated in several experiments investigating the perception of motion-in-depth. Some of the other observers may have had prior experience with psychophysical experiments investigating motion and depth perception. They were compensated at £5/hour for their time. All observers had normal or corrected to normal vision and passed the TNO test. The experimental procedures used were in accordance with the declaration of Helsinki and approved by the University of St. Andrews University Teaching and Research Ethics Committee (Ethics code: PS11472). All observers gave written informed consent.

### Eye movement analysis

Post-processing and data analysis were done in MATLAB^54^. First, blinks as detected by the eye tracker’s blink detector and all measurements flagged as’low quality’ by the eye tracker software were identified in each trial. If the number of those events was less than 15% of the number of data points in a trial, they were interpolated otherwise the whole trial was rejected. In the next step, horizontal vergence was computed from the eye position data as the difference between the left and right horizontal eye position. This resulted in convergence being represented by negative and divergence by positive vergence values (see Fig. 1). The resulting vergence trace was smoothed using a moving average filter with a span of 50 ms. To account for random vergence fluctuations during the prelude, the vergence position at the end of the prelude was subtracted from all subsequent vergence values so that each vergence trace started at 0° vergence. To identify vergence traces with extremely large vergence values, for example because of saccades, we looked at all trials collected for a specific condition, separately for each observer. At each time point of the vergence traces, the distribution of vergence values was determined. Traces that contained values outside of approximately ±2.7 *SD* at any time point were discarded.

### Analysis of vergence

The analysis of the vergence eye movements was based on each observers’ single trials. To determine the vergence velocity and the direction of the vergence eye movements (con- or divergence), we first identified the point on the vergence trace halfway between the minimum and maximum vergence values of the vergence trace in the interval from stimulus onset to onset +750 ms (see Fig. 10). Then we fit a line with a length of 100 ms through this point to the vergence trace. The slope of this line provided an estimate of the vergence velocity at this point.

For the RAMP motion stimulus, the slope of the stimulus was 2°/s. For the STEP stimulus, the slope is, by definition, undefined (Infinity). The sign of the slope indicates the direction of the vergence eye movement (positive: divergence, negative: convergence). Based on this analysis, we recorded the proportion of vergence eye movements whose direction coincided with the direction of the stimulus movement (towards/away). We refer to vergence eye movements that were in the direction expected by the stimulus movement, i.e., convergence for approaching and divergence for receding stimuli, as consistent vergence movements, and to their relative frequency as proportion consistent. As an alternative measure for analysing the vergence eye movements, we used the area under the vergence trace. The area was computed over the entire length of the vergence trace (2 s) by using trapezoidal numerical integration. This integral over position/displacement data is also known as “absement” and is a measure of sustained displacement from an initial position. The absement for the stimulus movements was the same for RAMP and STEP stimulus motion (4 deg · s) allowing a direct comparison of the vergence magnitudes for RAMPs and STEPs. Both methods resulted in a similar pattern of results.

## Supplementary information

Supplementary Information

## Data Availability

The data are available online from the Open Science Framework (https://osf.io/9up4e/).
