# Supplementary material for: Relative contributions to vergence eye movements of two binocular cues for motion-in-depth
Source: Sci Rep. 2019 Nov 22;9:17412. doi: 10.1038/s41598-019-53902-y (PMC6874608; doi:10.1038/s41598-019-53902-y)
Supplement: Supplementary file 1 — Supplementary Information [file 41598_2019_53902_MOESM1_ESM.pdf]

Relative contributions to vergence eye movements of two binocular cues for motion-in-depth

Martin Giesel[1,*], Alexandra Yakovleva[2], Marina Bloj[3], Alex R. Wade[4], Anthony M. Norcia[2], and Julie M. Harris[1]

[1]School of Psychology and Neuroscience, University of St Andrews, UK
[2]Department of Psychology, Stanford University, USA
[3]School of Optometry and Vision Sciences, University of Bradford, UK
[4]Department of Psychology, University of York, UK
[*]martin.giesel@abdn.ac.uk

Supplementary information

Additional methods

Generation of random-dot stereograms

Figure S1 shows schematically how random-dot stereograms that combine or isolate the two binocular cues are generated. This text follows the description in[1]. To create a FULL cue random-dot stereogram, each dot in one eye is paired with a dot of the same contrast in the other eye. The dots move with the same speed in opposite directions creating coherent monocular motion in each eye. Throughout the movement the dots remain at corresponding positions in the two eyes resulting in coherent cyclopean motion (a change in binocular disparity over time). Note that the dots in the FULL cue stimulus are correlated both spatially (across eyes) and temporally (across video frames).

A random-dot stereogram that isolates CD information (also referred to as a dynamic random-dot stereogram) is created by randomly repositioning dots in each video frame so that the changes in binocular disparity remain consistent while the temporal correlations between frames are removed so that there is no coherent monocular motion within each eye. Without consistent monocular motion in each eye, no IOVD cue is available.

A stimulus that isolates IOVD information must generate consistent monocular motion signals in the two eyes without giving rise to consistent changes in disparity. This can be achieved by using de-correlated or anti-correlated random-dot stereograms. De-correlated random-dot stereograms exploit the fact that for the computation of coherent disparity the visual system has to be able to match corresponding elements in the retinal images of the two eyes. This matching process is obstructed or disrupted if the spatial separation between elements in the two eyes becomes too large. In a dIOVD random-dot stereogram dots in one eye have no corresponding dots in the other eye so that the CD cue is minimised. There is, however, consistent dot motion within each of the two eyes[2,3].

The anti-correlated (aIOVD) random-dot stereogram resembles the FULL cue random-dot stereogram with the difference that each dot in one eye is paired with a dot of the opposite contrast in the other eye (inter-ocular contrast reversal), e.g., a black dot in the left eye is paired with a white dot at the corresponding position in the right eye[4]. The rationale for using aIOVD stimuli is that it has been found that perceived depth in static anti-correlated displays is weak or non-existent[5–9]. For a psychophysical comparison and discussion of aIOVD and dIOVD stimuli see[1].

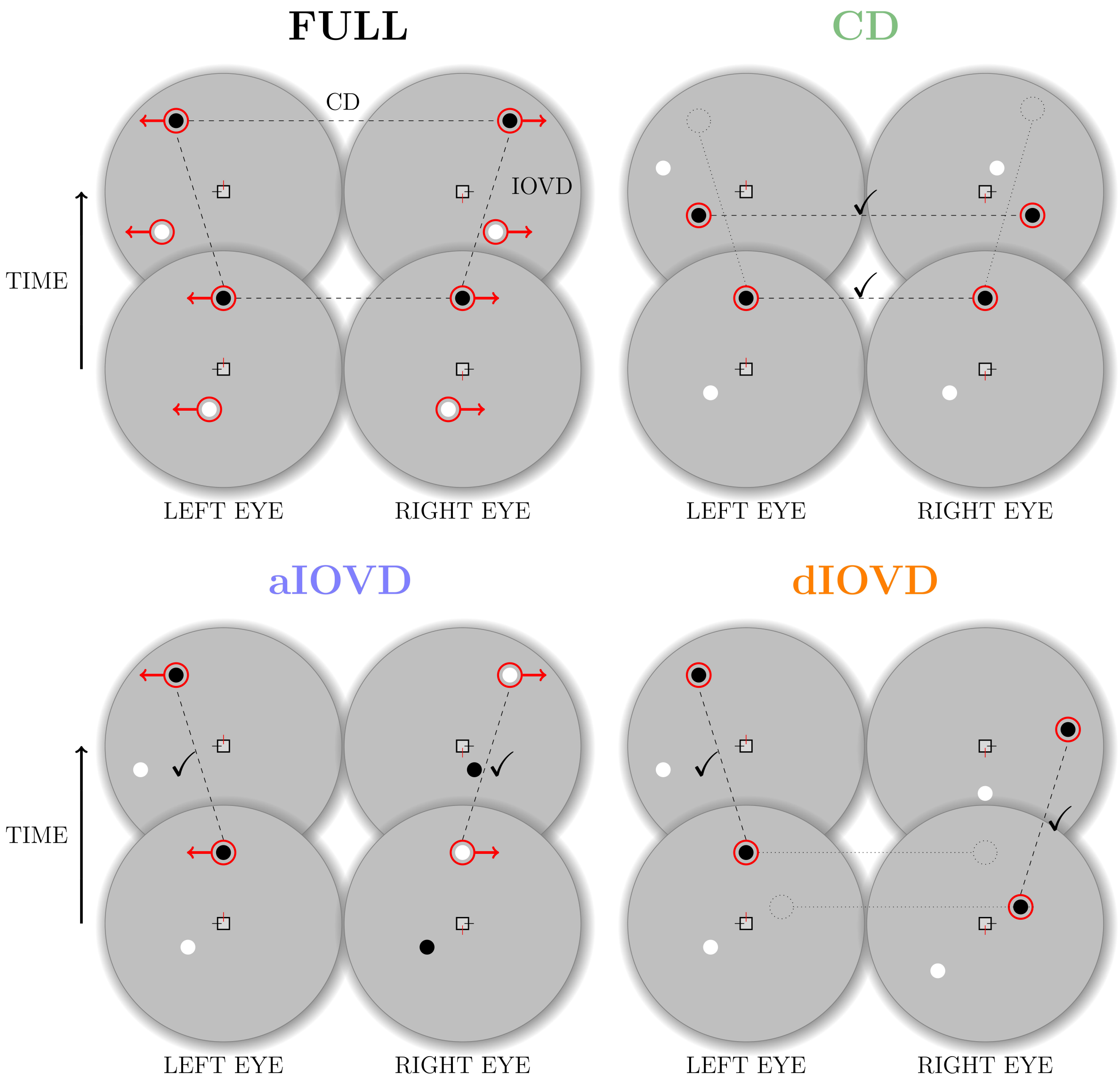

Figure S1. Schematic depiction of two consecutive frames of random-dot stereograms for FULL cue (top left), CD (top right), aIOVD (bottom left), and dIOVD (bottom right) stimuli (reprinted from[1]).

Number of trials per observer

Figures S2 and S3 show the number of trials that observers completed and the number of trials included in the analysis.

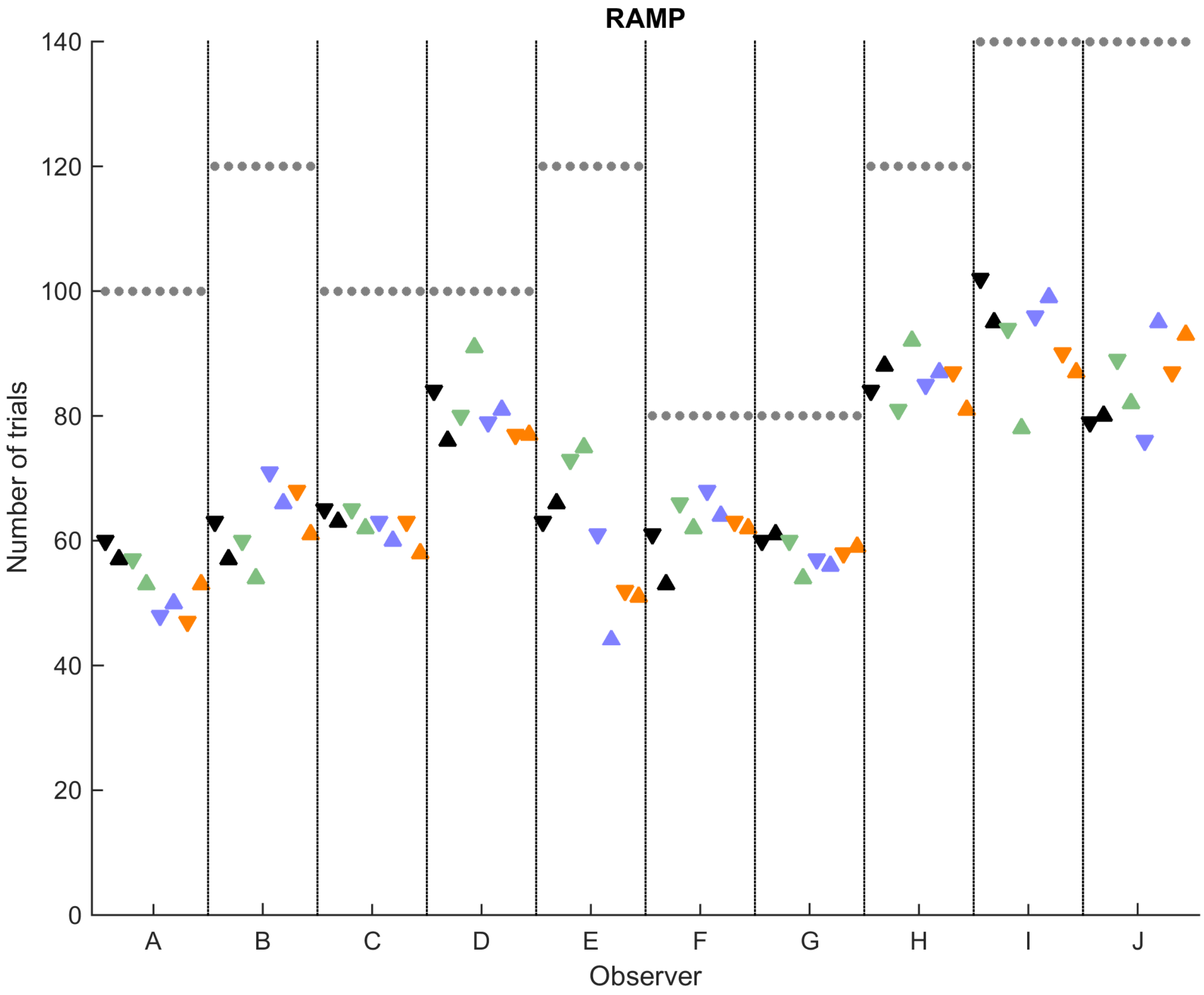

Figure S2. Number of usable trials for each observer and motion-in-depth condition for RAMP motion. Triangles indicate the number of usable trials. Black: FULL cue; green: CD; blue: aIOVD; orange: dIOVD. Upward triangles: motion away; downward triangles: motion towards. Gray filled circles indicate the number of trials completed by each observer per condition.

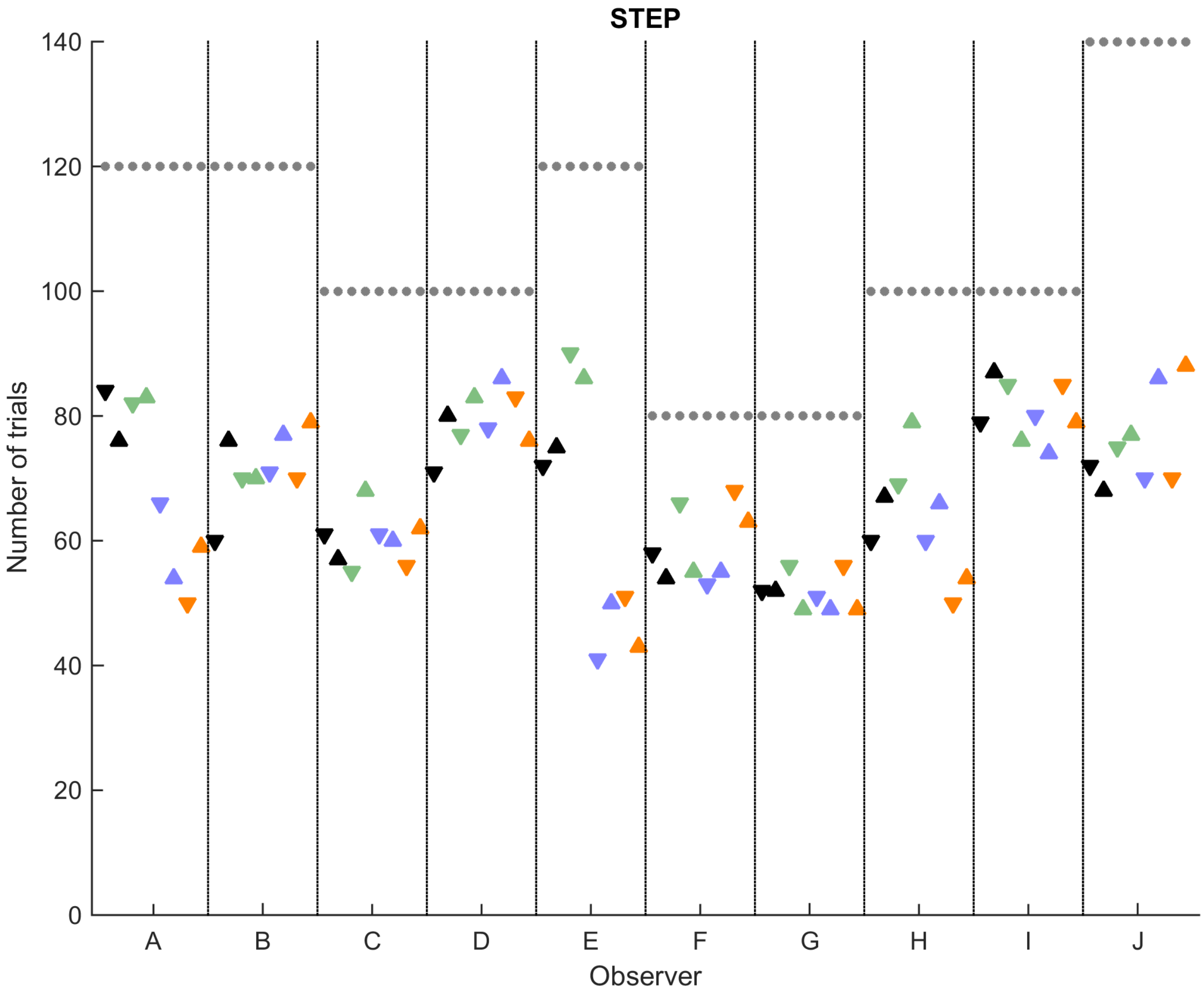

Figure S3. Number of usable trials for each observer and motion-in-depth condition for STEP motion. Triangles indicate the number of usable trials. Black: FULL cue; green: CD; blue: aIOVD; orange: dIOVD. Upward triangles: motion away; downward triangles: motion towards. Gray filled circles indicate the number of trials completed by each observer per condition.

Additional results

Probability density distributions

	RAMP				STEP			
	Towards		Away		Towards		Away	
Stimulus	M	s	M	s	M	s	M	s
FULL	-2.2411	1.1131	2.2145	1.3340	-4.6878	4.3243	1.8803	4.2555
CD	-2.1984	1.1824	2.0423	1.2844	-5.2327	3.6717	3.1417	4.0019
aIOVD	-0.1569	1.7916	0.4405	1.7377	-1.4865	2.6200	1.0648	3.0695
dIOVD	0.2522	1.7757	0.1338	1.7037	0.1342	2.3503	-0.0947	2.4614

Table S1. Mean and standard deviation for the density estimates of the slopes.

Average vergence traces

Figure S4 shows vergence traces averaged over trials for each observer classified based on the consistency of the slopes, i.e., for each observer, motion-in-depth stimulus and motion direction we averaged separately over consistent and inconsistent vergence traces. Looking at the consistent traces (first and last columns) for FULL cue and CD stimuli, reveals the different shapes of the vergence traces in response to RAMPs and STEPs. The inconsistent traces for these stimuli, do not show these shapes as clearly, although among the traces classified as inconsistent are some that probably have been classified incorrectly by our automatic procedure. Using the area under the vergence trace as measure results in fewer false classifications (see Figure S11), and shows even clearer that inconsistent vergence traces do not reflect these differences in the shape of the vergence trace characteristic for the stimuli. For IOVD stimuli, consistent traces for RAMPs and STEPs resembled each other and were similar to the inconsistent traces for most observers with the exception of observer J. The traces for FULL cue and CD stimuli seem to have a common delay after stimulus onset, whereas for IOVD stimuli the delay seems to be shorter and more variable.

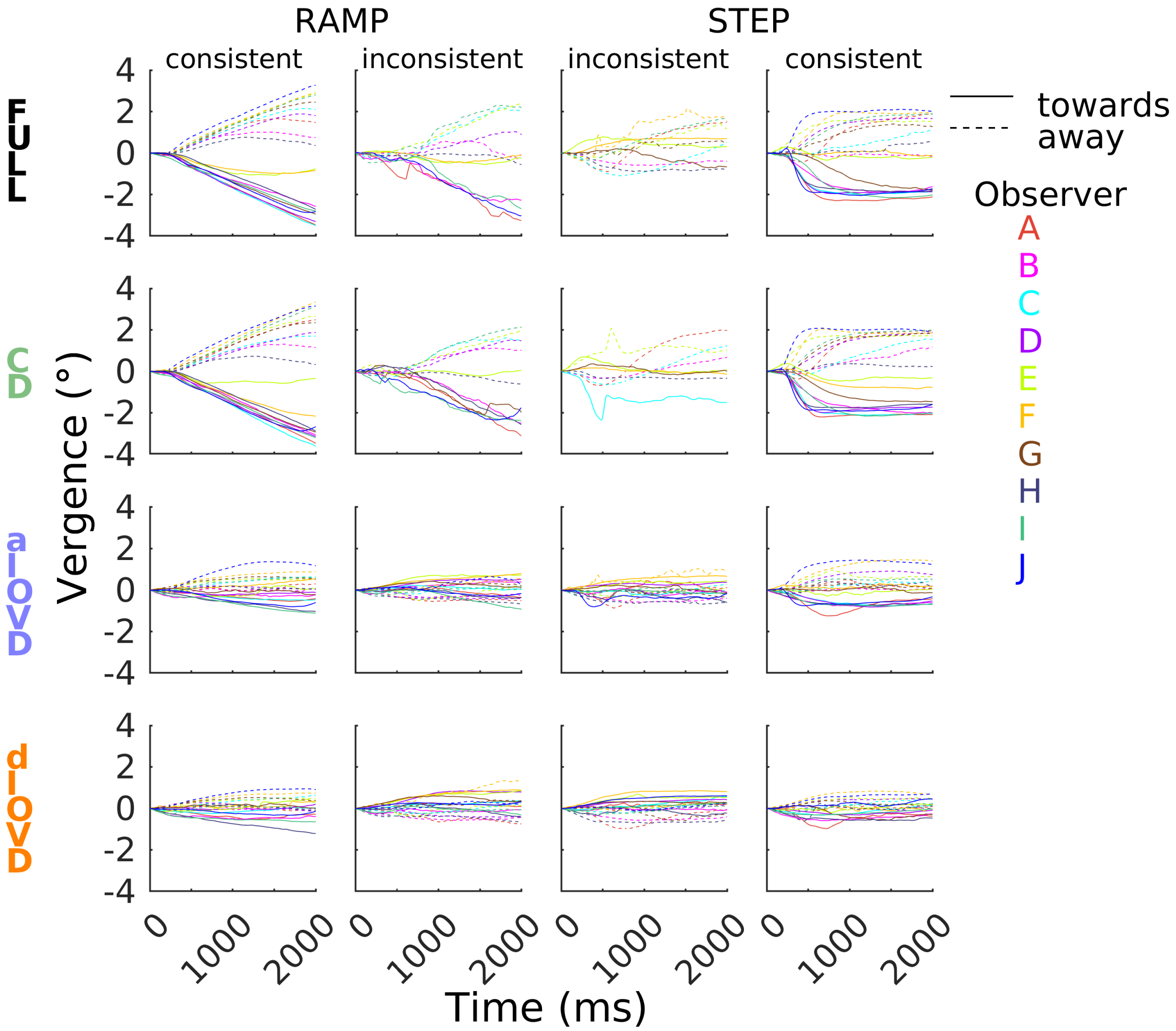

Figure S4. Vergence traces averaged over trials for each observer. The x-axes show time in ms (starting from stimulus onset), and the y-axes vergence in degrees. Solid vergence traces represent responses to approaching stimuli, and dashed traces responses to receding stimuli. Different colours indicate different observers. The first row shows vergence for FULL cue, the second for CD, the third for aIOVD, and the fourth for dIOVD. The first two columns show consistent and inconsistent responses to RAMP motion, and the last two columns inconsistent and consistent responses to STEP stimuli.

Vergence analysis based on the area-under-the-curve

In the following, we show result of the data analysis based on the area under the vergence traces. The figures are analog to the figures showing slopes in the main paper.

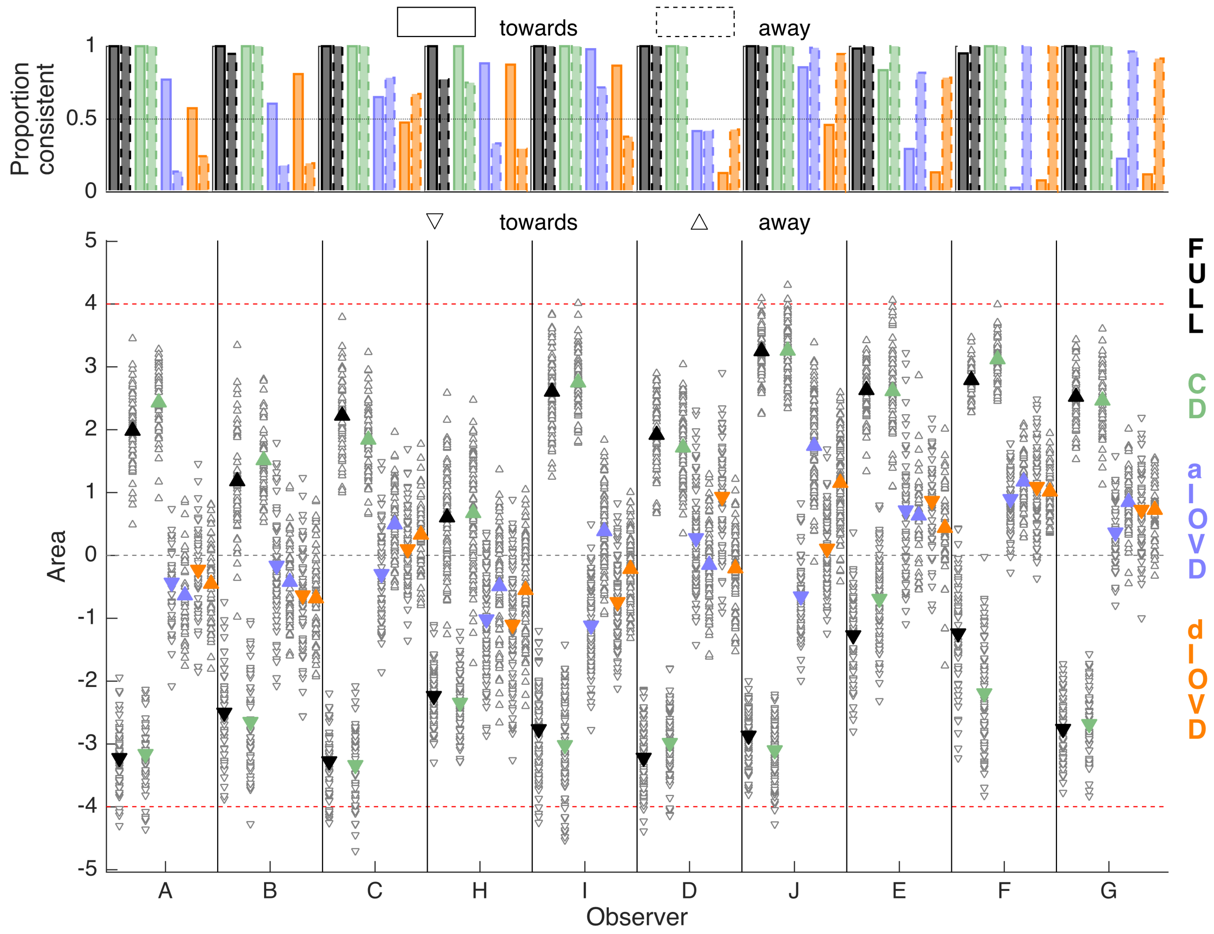

Figure S5. Proportion of consistent area signs (top), area magnitudes and signs (bottom) for the RAMP condition separately for each observer, type of motion-in-depth stimulus and motion direction. The x-axis shows the different observers. The symbol/bar colour indicates the stimulus types (Black: FULL; green: CD; blue: aIOVD; orange: dIOVD). (Bottom) The y-axis shows the area under the vergence traces in deg · s. Negative areas indicate convergence, positive areas divergence. Open grey symbols show areas for single trials, and filled coloured symbols represent the average over the single trial areas. Upward triangles indicate stimulus motion away and downward triangles stimulus motion towards. The red dashed lines indicate the area under the RAMP stimulus (±4 deg · s). (Top) The y-axis shows the proportion of consistent area signs. Bars with a solid edge represent stimulus motion towards, and bars with a dashed edge represent results for stimulus motion away.

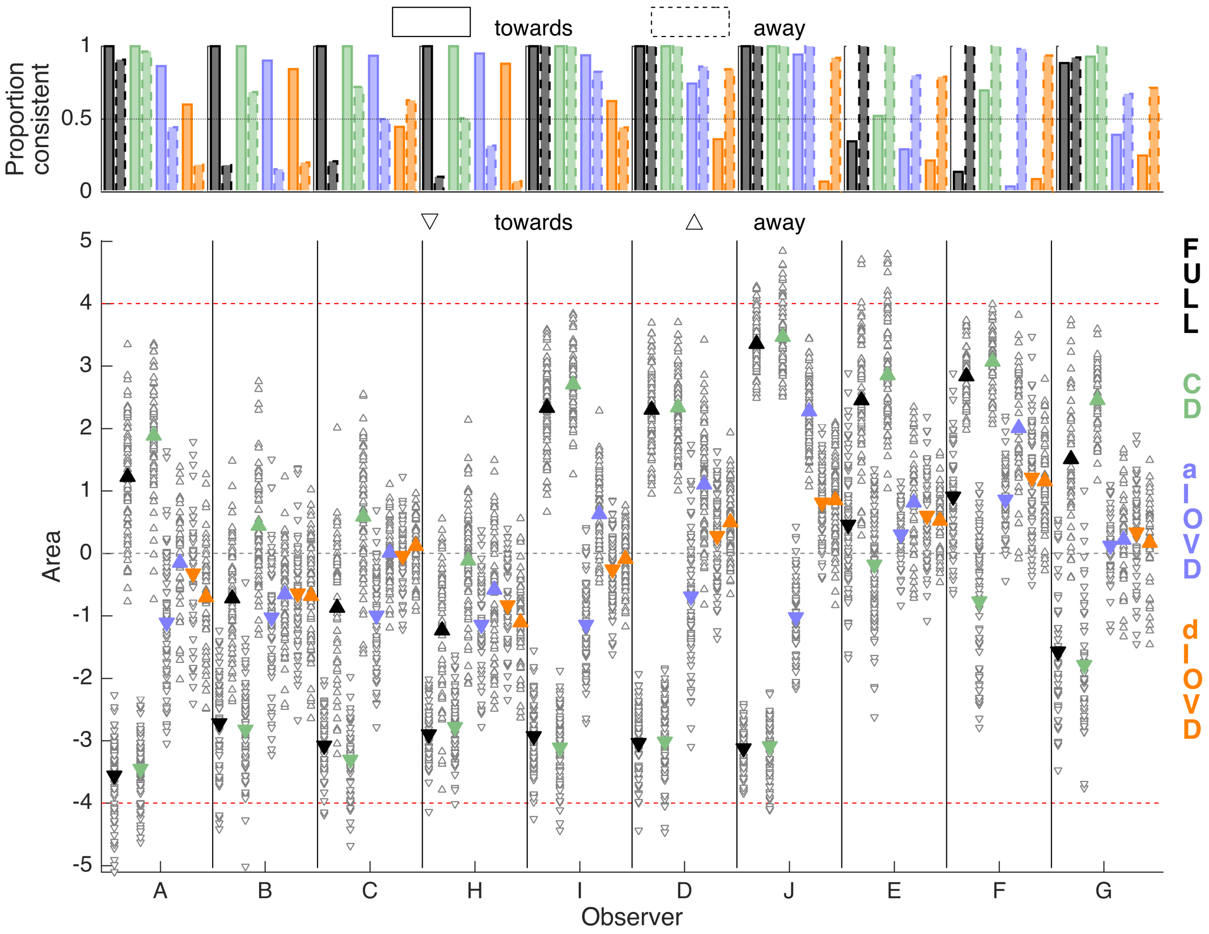

Figure S6. Proportion of consistent area signs (top), area magnitudes and signs (bottom) for the STEP condition separately for each observer, type of motion-in-depth stimulus and motion direction. The x-axis shows the different observers. The symbol/bar colour indicates the stimulus types (Black: FULL; green: CD; blue: aIOVD; orange: dIOVD). (Bottom) The y-axis shows the area under the vergence traces in deg · s. Negative areas indicate convergence, positive areas divergence. Open grey symbols show areas for single trials, and filled coloured symbols represent the average over the single trial areas. Upward triangles indicate stimulus motion away and downward triangles stimulus motion towards. The red dashed lines indicate the area under the STEP stimulus (±4 deg · s). (Top) The y-axis shows the proportion of consistent area signs. Bars with a solid edge represent stimulus motion towards, and bars with a dashed edge represent results for stimulus motion away.

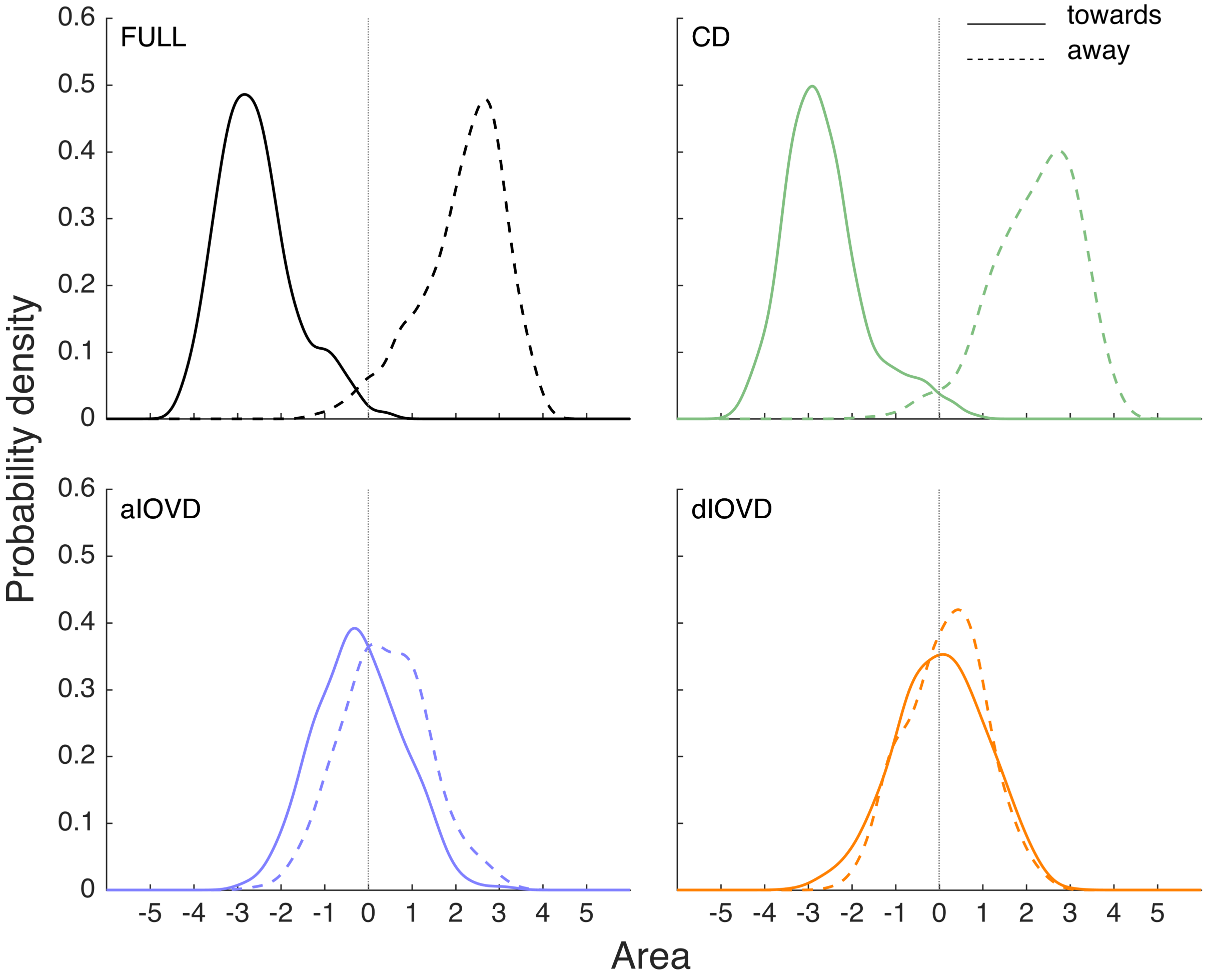

Figure S7. Area density estimates for RAMP motion stimuli. The x-axis shows the area in deg · s, and the y-axis the probability density. The areas from all observers and trials have been pooled. Negative areas represent convergence, positive areas divergence. Distributions with a solid outline represent stimulus motion towards, and distributions with a dashed outline represent stimulus motion away. The total number of areas differed between conditions.

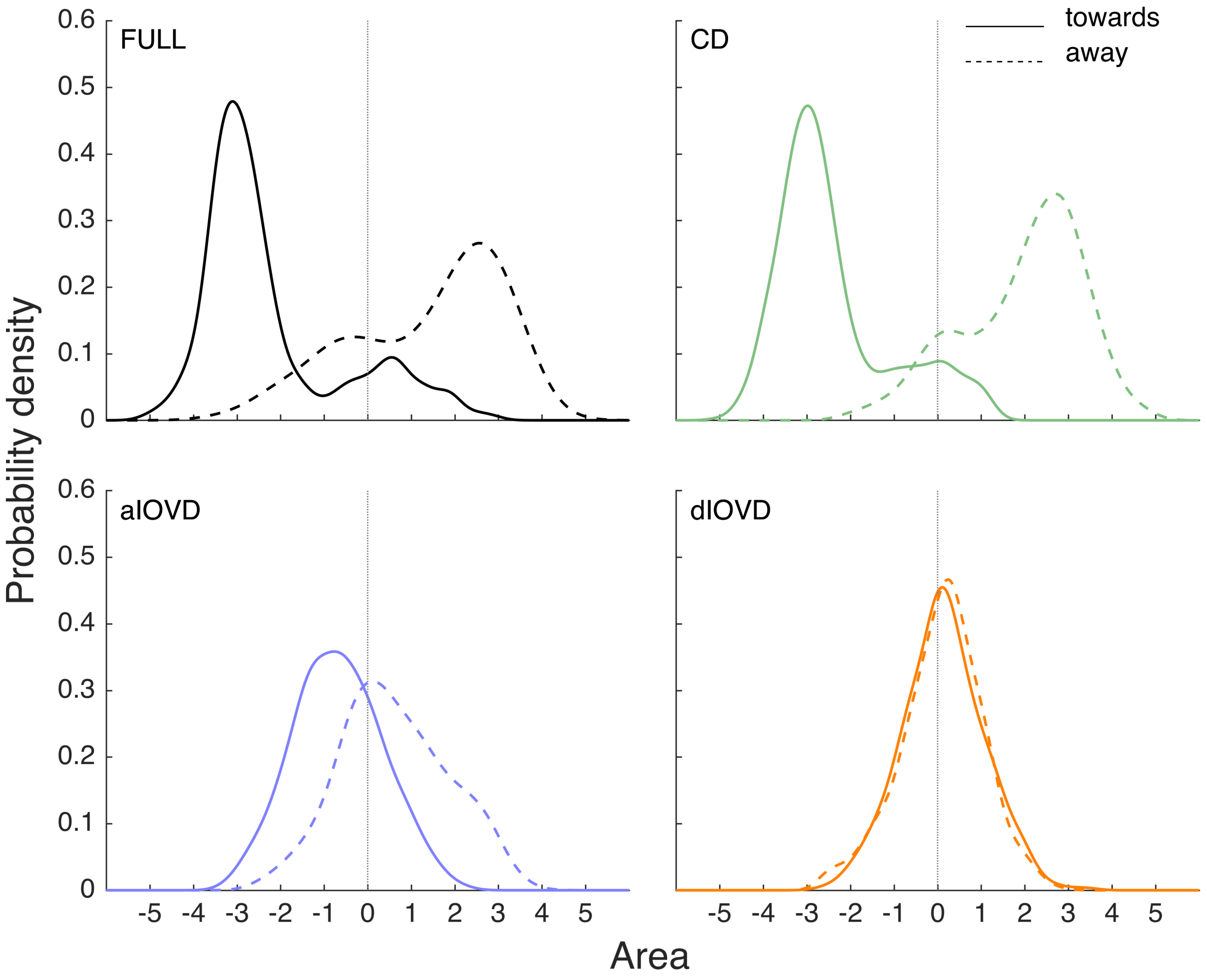

Figure S8. Area density estimates for STEP motion stimuli. The x-axis shows the area in deg · s, and the y-axis the probability density. The areas from all observers and trials have been pooled. Negative areas represent convergence, positive areas divergence. Distributions with a solid outline represent stimulus motion towards, and distributions with a dashed outline represent stimulus motion away. The total number of areas differed between conditions.

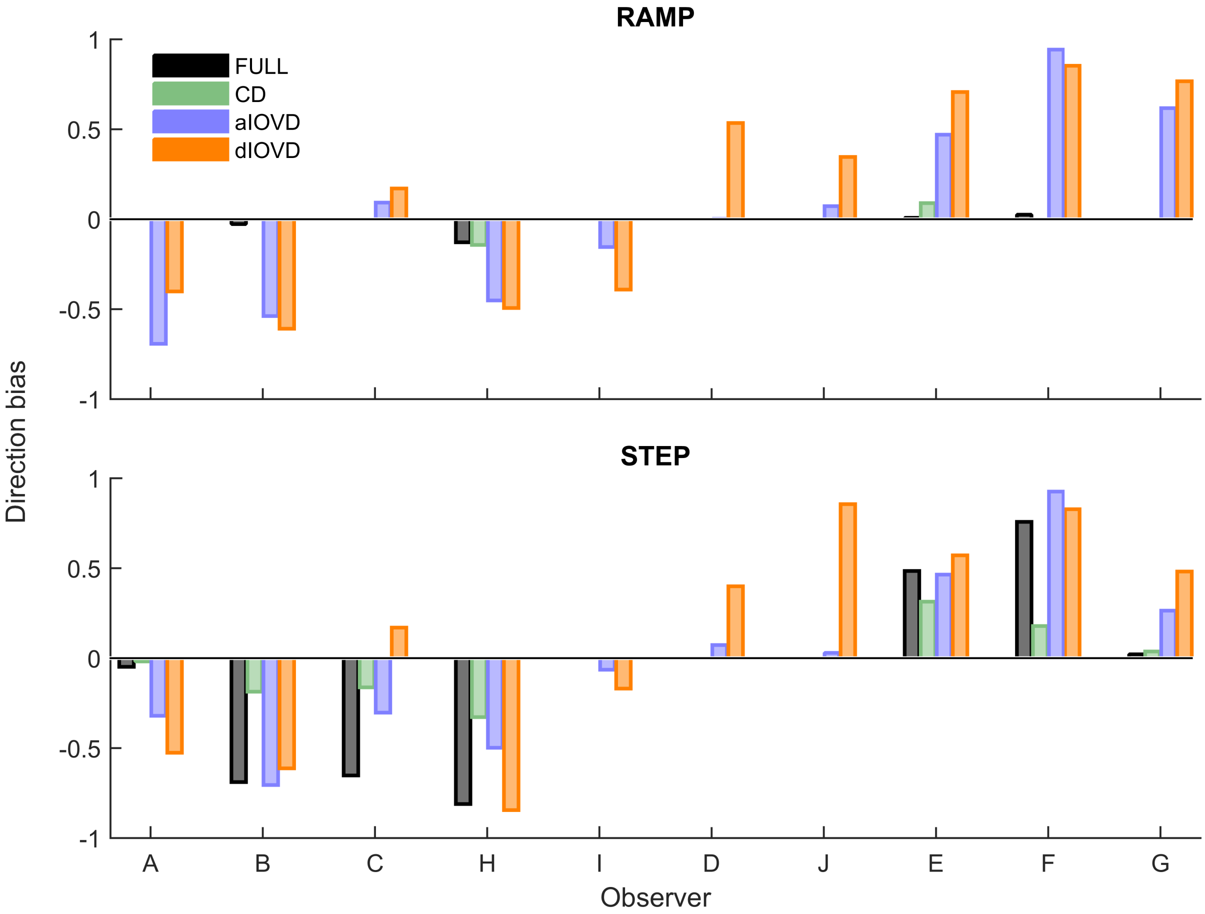

Figure S9. Direction bias for RAMP (top) and STEP (bottom) motion. The x-axis shows the observers, the y-axis the direction bias. A negative bias indicates a preference for convergence, and a positive bias indicates a preference for divergence. Different bar colours represent the different types of motion-in-depth stimuli (Black: FULL; green: CD; blue: aIOVD; orange: dIOVD). See text for how the bias was computed.

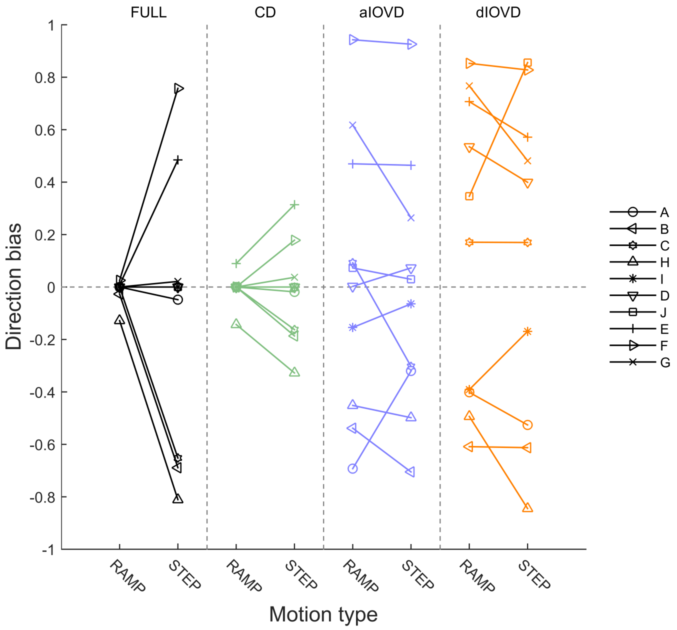

Figure S10. Direction bias for RAMP and STEP motion. The x-axis shows the motion type, and the y-axis the direction bias. A negative bias indicates a preference for convergence, and a positive bias indicates a preference for divergence. Individual lines represent different observers. Different stimulus types are shown in separate columns (Black: FULL; green: CD; blue: aIOVD; orange: dIOVD). See text for how the bias was computed.

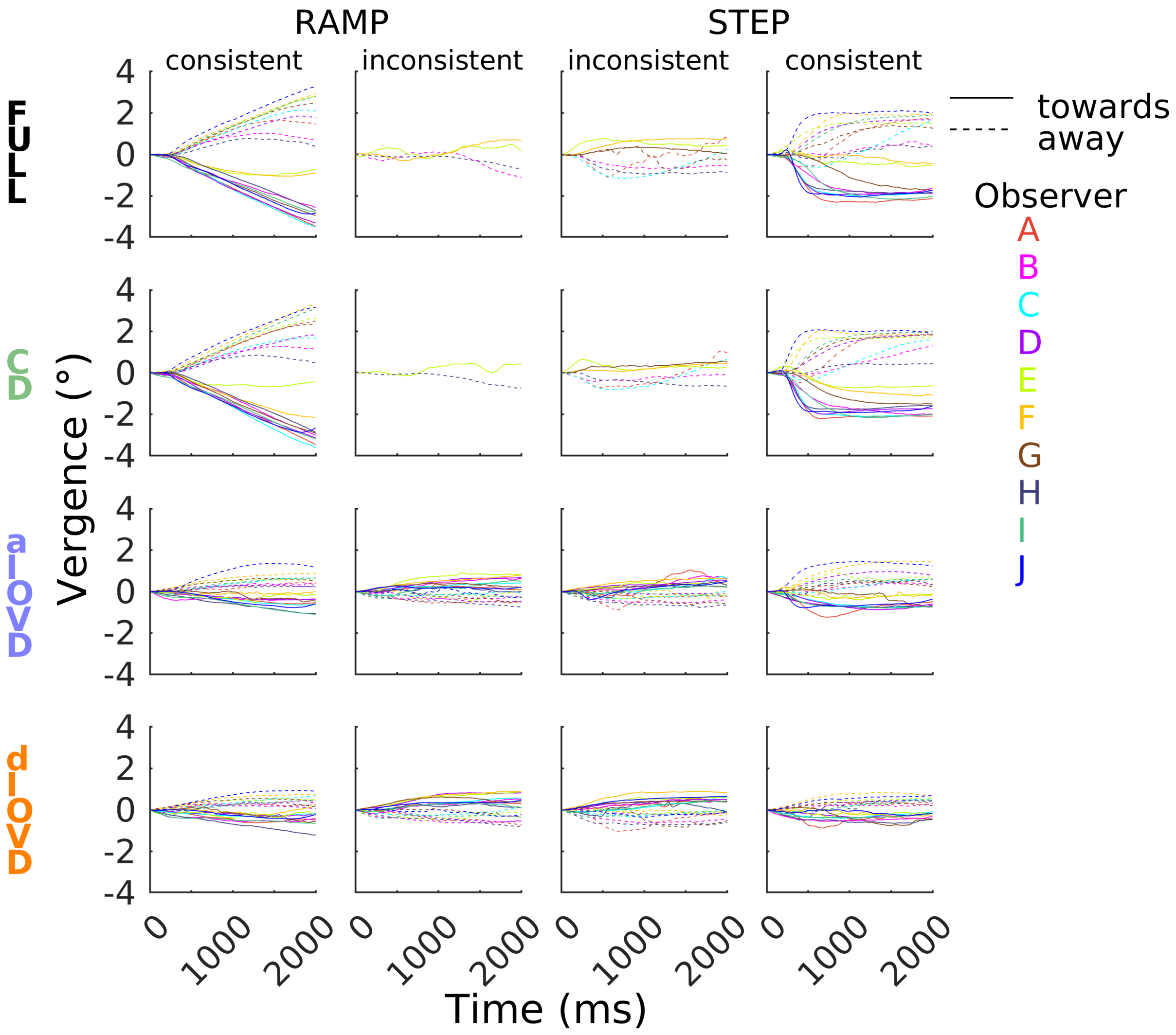

Figure S11. Mean vergence traces for each observer. The x-axes show time in ms, and the y-axes vergence in degrees. Solid vergences traces represent responses to approaching stimuli, and dashed vergence traces show responses to receding stimuli. Different colours indicate different observers. The first row shows vergence for FULL, the second for CD, the third for aIOVD, and the fourth for dIOVD. The first two columns show consistent and inconsistent responses to RAMP motion, and the last two columns inconsistent and consistent responses to STEP stimuli.

Lateral motion control experiment

To determine the direction into which a random-dot stereogram moved based on mononcular motion signals, the visual system has to match dots between frames. In a dense random-dot stereogram, this match might be ambiguous. The frequency of such false matches between frames, depends on the density of the random-dot stereogram and the size of the step. With a single dot there can be no false matches. If the step size is small, e.g., only one pixel per frame, the distance between the position of a dot in the current and the preceding frame will be small and probably smaller than possible false matches assuming that matching roughly follows a nearest neighbor metric. However, if the dots in a dense random-dot stereogram perform a large step, the frequency of other dots being closer to the position of a dot in the preceding frame than its current position, might result in ambiguous motion signals or even in a reversal of the perceived motion direction. With RAMP motion, which consists of several small steps, this is less likely to happen.

We conducted a control experiment to determine whether STEP motion as used in our experiment generated a consistent motion direction signal. Two observers, who participated in the main experiment (F and J), were presented with random-dot stereograms moving laterally (leftwards or rightwards) instead of in depth. Apart from this change in the movement direction all other stimulus and presentation parameters were identical to those in the main experiment. Particularly, observers were presented with all four types of random-dot stereograms (FULL cue, CD, aIOVD, and dIOVD) and both types of motion (RAMP and STEP). Each stimulus condition was presented 40 times. The stimuli were viewed binocularly through the 3D glasses. Observers were asked to decide whether the dots moved to the left or to the right by pressing the appropriate key. In this experiment, we were only interested in the perceptual responses, so no eye movements were recorded. Stimulus types were interleaved and motion types blocked. Figure S12 shows the result of the lateral motion control experiment. For RAMP motion, both observers almost always identified the correct motion direction for FULL cue, aIOVD, and dIOVD stimuli. The chance level performance for the CD stimulus is expected because the CD stimulus is designed not to contain a consistent monocular motion signal. For STEP motion, both observers performed for all types of stimuli at or near chance level. This indicates that STEP motion with random-dot stereograms as used in our experiments did not contain a consistent motion signal.

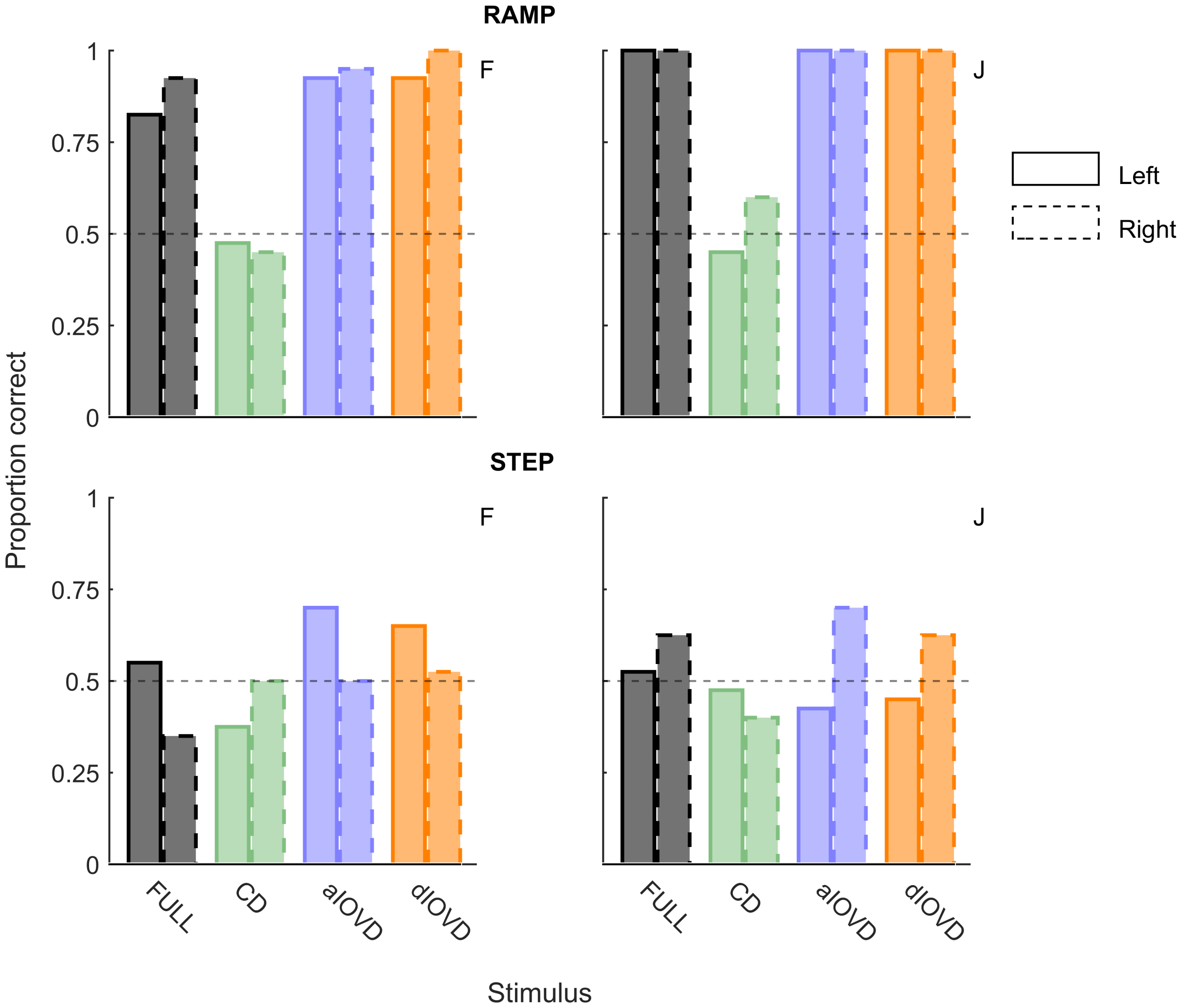

Figure S12. Results of the lateral motion control experiment. The x-axis shows the four types of random-dot stimuli and the y-axis proportion correct. Bars with solid lines indicate leftward motion and bars with dashed lines rightward motion. The first row shows results for RAMP motion and the second row for STEP motion. The first column shows results for observer F and the second for observer J.

Perceptual responses

In the experiments, the observers had the task to decide whether the random-dot stereograms moved towards or away from them. Figures S13 and S14 show the proportion of correctly detected directions of motion-in-depth separately for each observer and motion-direction.

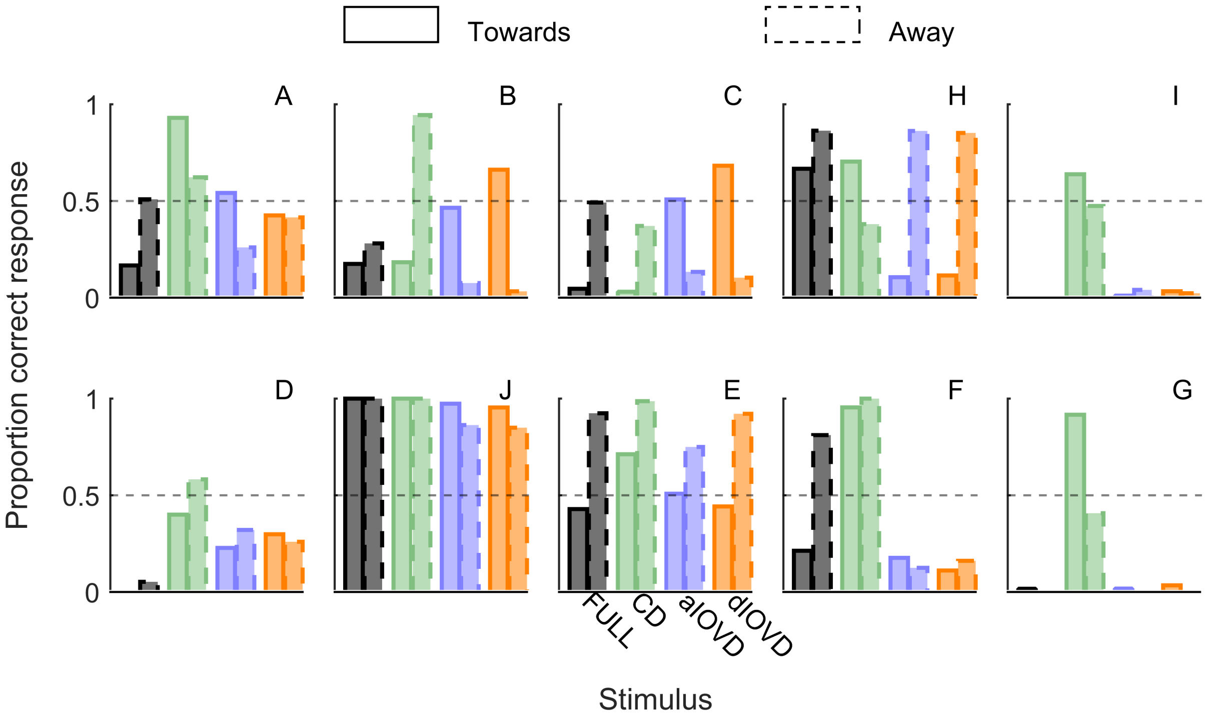

Figure S13. Proportion of correctly detected directions of motion-in-depth for RAMPs separately for each observer and motion direction. The x-axis shows the different types of stimuli, the y-axis proportion correct. Black: FULL; Green: CD; Blue: aIOVD; Orange: dIOVD.

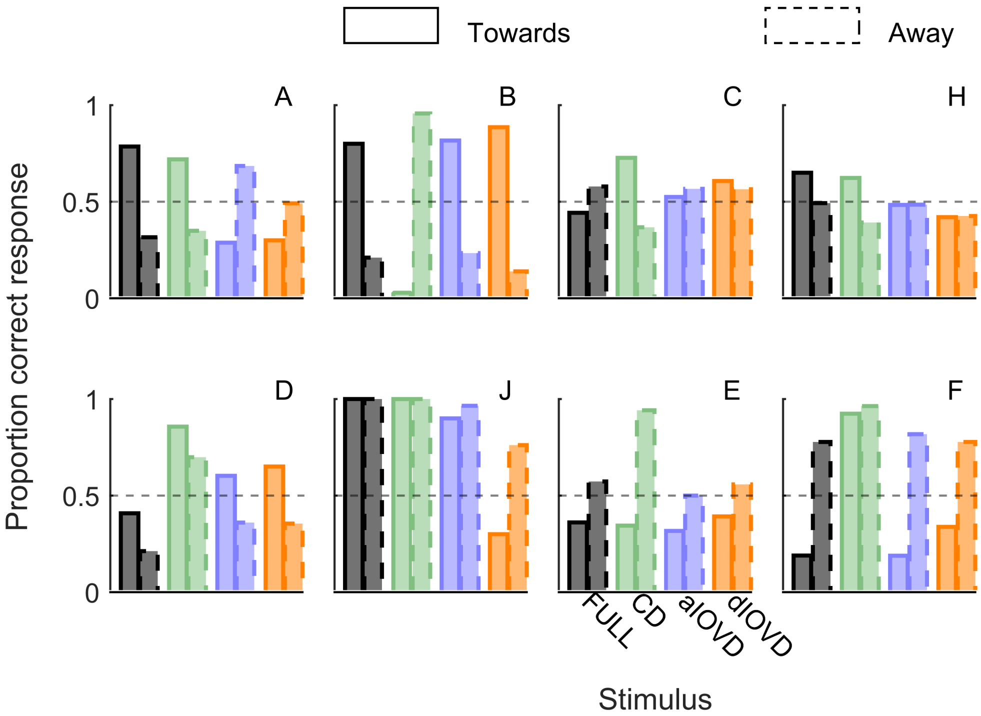

Figure S14. Proportion of correctly detected directions of motion-in-depth for STEPs separately for each observer and motion direction. The x-axis shows the different types of stimuli, the y-axis proportion correct. Black: FULL; Green: CD; Blue: aIOVD; Orange: dIOVD.

To analyse the relation between vergence eye movements and the perceptual discrimination of motion-in-depth, Figures S15 and S16 show plots of the proportion of consistent slopes against the proportion of correct perceptual responses.

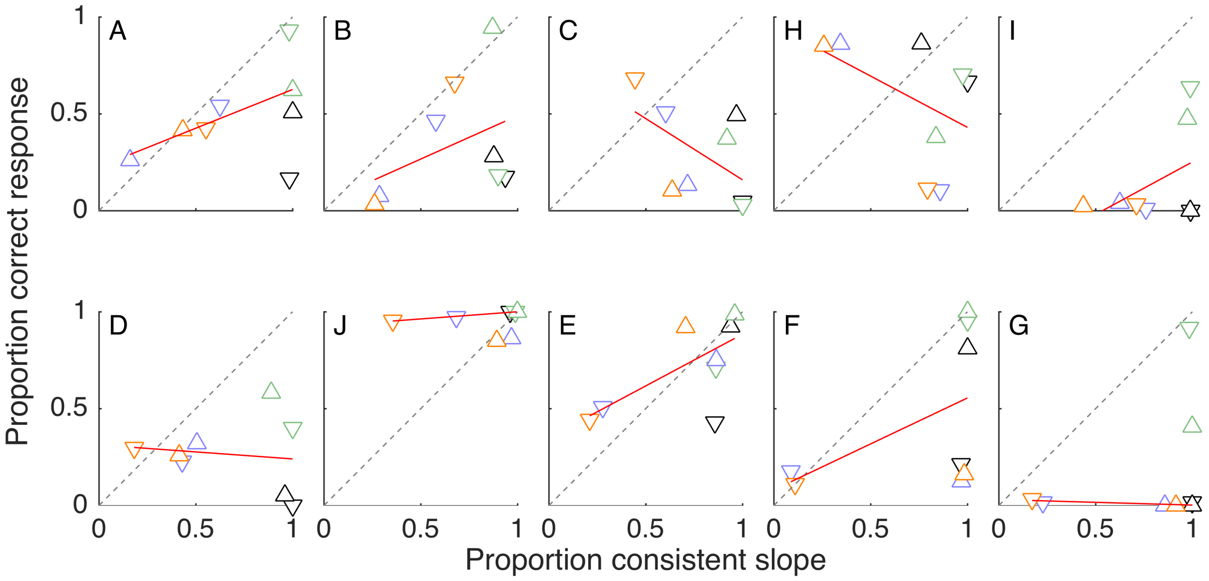

Figure S15. Comparison of slope consistency and perceptual response for RAMPs separately for each observer and motion direction. The x-axis shows the proportion of consistent slopes, the y-axis proportion of correct perceptual responses. Black: FULL; Green: CD; Blue: aIOVD; Orange: dIOVD. Downward triangles: towards; upward triangles: away. The red line is a robust regression fit to the combined data points.

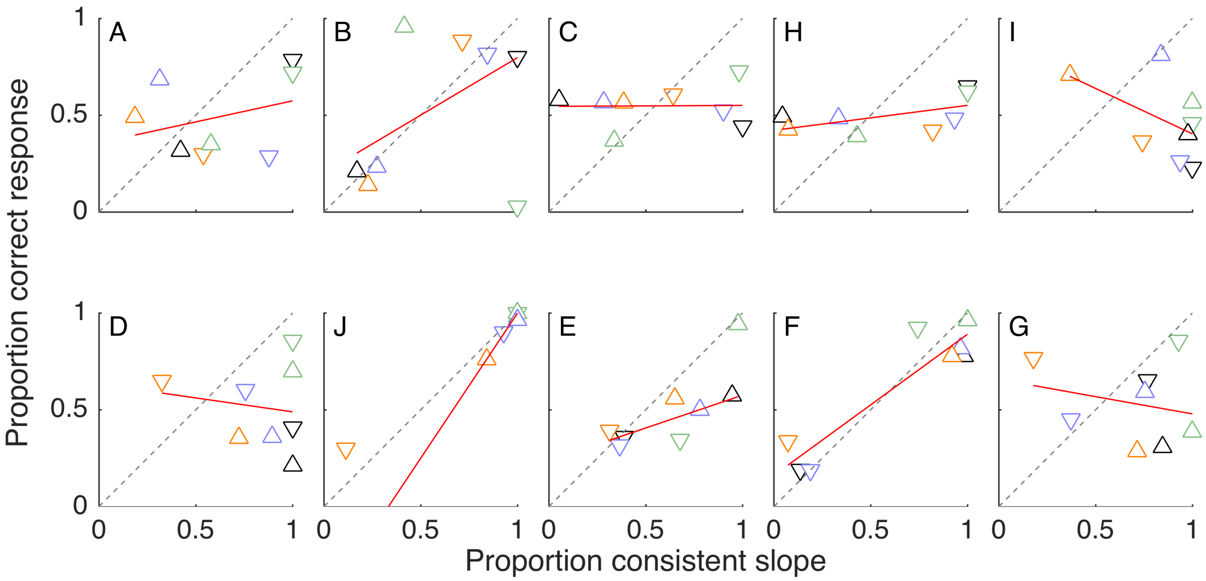

Figure S16. Comparison of slope consistency and perceptual response for STEPs separately for each observer and motion direction. The x-axis shows the proportion of consistent slopes, the y-axis proportion of correct perceptual responses. Black: FULL; Green: CD; Blue: aIOVD; Orange: dIOVD. Downward triangles: towards; upward triangles: away. The red line is a robust regression fit to the combined data points.

References

1. Giesel, M., Wade, A. R., Bloj, M. & Harris, J. M. Investigating human visual sensitivity to binocular motion-in-depth for anti- and de-correlated random-dot stimuli. *Vis.* **2** (2018). URL http://www.mdpi.com/2411-5150/2/4/41. DOI 10.3390/vision2040041.

2. Allison, R., Howard, I. & Howard, A. Motion in depth can be elicited by dichoptically uncorrelated textures. *Percept. ECVP abstract* **27**, 46 (1998).

3. Shioiri, S., Saisho, H. & Yaguchi, H. Motion in depth based on inter-ocular velocity differences. *Vis. Res.* **40**, 2565–2572 (2000).

4. Rokers, B., Cormack, L. K. & Huk, A. C. Strong percepts of motion through depth without strong percepts of position in depth. *J. Vis.* **8**, 6.1–610 (2008). DOI 10.1167/8.4.6.

5. Cogan, A. I., Kontsevich, L. L., Lomakin, A. J., Halpern, D. L. & Blake, R. Binocular disparity processing with opposite-contrast stimuli. *Percept.* **24**, 33–47 (1995). DOI 10.1068/p240033.

6. Cogan, A. I., Lomakin, A. J. & Rossi, A. F. Depth in anticorrelated stereograms: effects of spatial density and interocular delay. *Vis. Res.* **33**, 1959–1975 (1993).

7. Cumming, B. G., Shapiro, S. E. & Parker, A. J. Disparity detection in anticorrelated stereograms. *Percept.* **27**, 1367–1377 (1998). DOI 10.1068/p271367.

8. Harris, J. M. & Rushton, S. K. Poor visibility of motion in depth is due to early motion averaging. *Vis. Res.* **43**, 385–392 (2003).

9. Julesz, B. *Foundations of cyclopean perception* (University of Chicago Press, Chicago, IL, 1971).
